# Two end-member earthquake preparations illuminated by foreshock activity on a meter-scale laboratory fault

**DOI:** 10.1038/s41467-021-24625-4

**Published:** 2021-07-14

**Authors:** Futoshi Yamashita, Eiichi Fukuyama, Shiqing Xu, Hironori Kawakata, Kazuo Mizoguchi, Shigeru Takizawa

**Affiliations:** 1 National Research Institute for Earth Science and Disaster Resilience, Tsukuba, Japan; 2 Department of Civil and Earth Resources Engineering, Kyoto University, Kyoto, Japan; 3 Department of Earth and Space Sciences, Southern University of Science and Technology, Shenzhen, China; 4 College of Science and Engineering, Ritsumeikan University, Kusatsu, Japan; 5 Central Research Institute of Electric Power Industry, Abiko, Japan

**Keywords:** Natural hazards, Geophysics, Seismology

## Abstract

The preparation process of natural earthquakes is still difficult to quantify and remains a subject of debate even with modern observational techniques. Here, we show that foreshock activity can shed light on understanding the earthquake preparation process based on results of meter-scale rock friction experiments. Experiments were conducted under two different fault surface conditions before each run: less heterogeneous fault without pre-existing gouge and more heterogeneous fault with pre-existing gouge. The results show that fewer foreshocks occurred along the less heterogeneous fault and were driven by preslip; in contrast, more foreshocks with a lower *b* value occurred along the more heterogeneous fault and showed features of cascade-up. We suggest that the fault surface condition and the stress redistribution caused by the ongoing fault slip mode control the earthquake preparation process, including the behavior of foreshock activity. Our findings imply that foreshock activity can be a key indicator for probing the fault conditions at present and in the future, and therefore useful for assessing earthquake hazard.

## Abstract (web summary)

By tuning fault surface heterogeneity, the authors report earthquake preparation processes respectively driven by preslip and cascade-up on a meter-scale laboratory fault. The findings suggest that foreshock activity can be useful for predicting when and how the impending mainshock will occur.

## Introduction

Regarding the earthquake preparation process on a fault surface, two end-member models have been proposed^1^. One is the preslip model: quasi-static slow slip initiates first, the slipped area expands at an accelerated rate, and it eventually leads to an unstable fast rupture over the whole fault area. This process has been well studied in theory, modeling^2–6^, and laboratory experiments^7–12^. The other one is the cascade-up model: one small earthquake spontaneously occurs, its stress transfer triggers another larger earthquake, and eventually the mainshock is triggered^1,13^. So far much debate has been made on which model can better explain the observed foreshock activity and whether the characteristics of foreshocks are related to those of the mainshock^1,14–16^. On the other hand, an actual earthquake preparation process may involve both models and the prevailing one can depend on the detailed fault conditions, such as the distribution of asperities and frictional properties^5,17^. For convenience, we extend the definition of the cascade-up model from its original form^1^, by allowing for concurrent slow slip and foreshocks but without the expansion of a single prominent slow slip patch. This can avoid the unresolved debate on the existence of aseismic slip^15,16^, and will facilitate our subsequent comparison.

In addition to natural observations, acoustic emission and foreshock activity have been extensively studied in the laboratory^18,19^. One main purpose of those studies was to understand the physical process toward the macroscopic failure. Especially, various studies reported that the *b* value, which represents the relative frequency-size distribution of seismicity, decreased toward the macroscopic failure and recovered afterward^20–24^. Foreshocks triggered by preslip were observed on a large-scale laboratory fault^8^, which might be an analog to the foreshock sequence preceding some natural earthquakes^25–27^. The cascade-up process thought to be assisted by preslip was also observed in the laboratory^9,17^, but the related details such as the relation between seismic activity and local stress on the fault, the statistical characteristics of seismic events, or the influence of fault surface condition were not reported there.

In the current study, we prepare two different types of surface condition on a meter-scale laboratory fault, contrasted by the degree of heterogeneity and gouge configuration. We investigate the preparation process, including the evolutions of fault stress and foreshock activity, toward the mainshock under each condition. Finally, we elucidate the connection between fault surface condition and foreshock activity, and further highlight the potential of foreshocks for assessing earthquake hazard.

## Results

### Experimental setup and basic results

We conducted rock friction experiments using a large-scale shear apparatus (Supplementary Fig. 1a). This apparatus uses a large-scale shaking table as the driving force to apply shear load to the simulated fault, which enables us to use a meter-scale rock specimen. We used a pair of rectangular metagabbro blocks as the experimental specimens. The metagabbro blocks were sampled from Tamil Nadu, south India. The nominal contact area (fault area) was 1.5-m long and 0.1-m wide. This large laboratory fault can be used to simulate different types of fault surface condition. It also allows us to monitor the ongoing process on the fault in detail by dense measurement arrays: 32 triaxial rosette strain gauges and 64 piezoelectric (PZT) acoustic sensors (Supplementary Fig. 1b, c). See “Methods” for the detailed methodology of the experiment.

We repeatedly conducted experiments with the same pair of rock specimens (Supplementary Table 1), causing an evolution of fault surface condition^28^. To minimize the impact of the total cumulative displacement, we focus on two experiments successively conducted under the same loading condition (normal stress of 6.7 MPa, constant loading rate of 0.01 mm/s, and total slip amount of ~7 mm) but with different fault surface conditions before each run: the experiment LB12-011 preserved the gouge generated from the previous experiment under fast-rate (1 mm/s) and long-slip (~400 mm) loading, while the experiment LB12-012 started with all gouge removed. Note that the distribution of pre-existing gouge (PEG) for LB12-011 was heterogeneous, because we did not manipulate the generation or distribution of fault gouge. Effectively, LB12-012 represented a less heterogeneous (LH) condition and is referred to as “LH without PEG”, while LB12-011 represented a more heterogeneous (MH) condition and is referred to as “MH with PEG”.

**Fig. 1:**
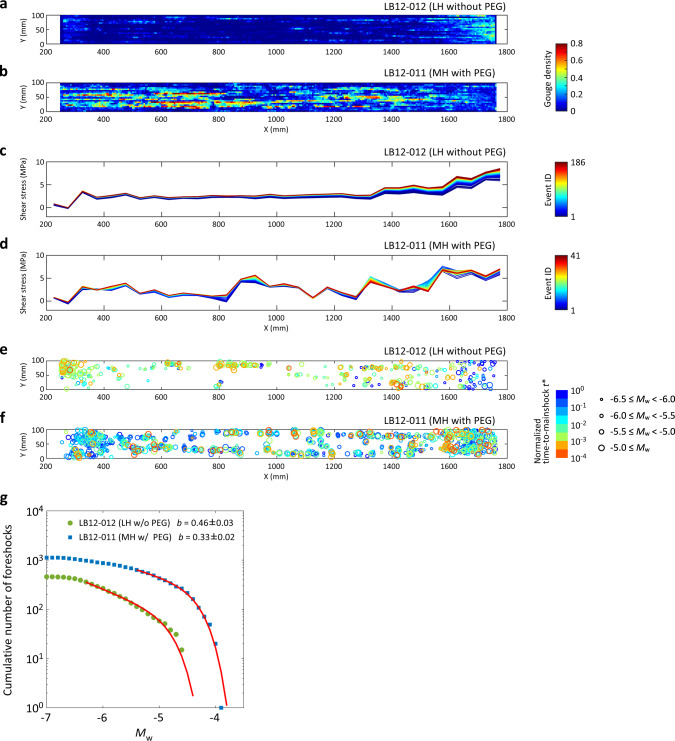
Distributions of gouge, shear stress, and foreshocks. Distribution of gouge after the experiment **a** LH without PEG (LB12-012) and **b** MH with PEG (LB12-011). The distribution map was estimated from the image analysis of pictures taken just after each experiment (the original pictures are shown in Supplementary Fig. 3). Note that the distribution of gouge shown in (**b**) was dominated by the PEG generated from an earlier test (LB12-010). The color of grid indicates the gouge density and procedure for constructing these images can be found in “Methods”. Distribution of local shear stress estimated from the strain gauge array just before each stick-slip event for **c** LH without PEG and **d** MH with PEG. The color of lines indicates event ID. Hypocenters of foreshocks for **e** LH without PEG and **f** MH with PEG. The size of circles scales with the moment magnitude (*M*_w_) of foreshocks. The color of circles indicates the normalized time-to-mainshock t* (see main text). **g** Frequency-size distribution of foreshocks for the two experiments. The estimated *b* value, moment magnitude of completeness, and corner moment magnitude, under the assumption that the detected foreshocks obey a TGR distribution, are listed in Supplementary Table 2. Red lines show the best-fit curves to a TGR distribution.

Difference in gouge distribution between the two experiments is shown by Fig. 1a, b, and Supplementary Fig. 3 (see also Supplementary Fig. 2 as a gray-scale version of Fig. 1): indicating more gouge patches over the entire length of the fault for MH with PEG than LH without PEG. Note that the gouge distributions before and after the experiment LB12-011 (Fig. 1b) were almost the same (Supplementary Fig. 3g, h), due to the small amount of total slip (~7 mm) during LB12-011 (Supplementary Table 1). Difference in local stress between the two experiments is shown by Fig. 1c, d: indicating a stronger spatial heterogeneity of local shear stress for MH with PEG than LH without PEG.

Clear differences between the two experiments can also be observed in other aspects (Supplementary Fig. 4a, b). While both experiments have hosted many stick-slip events, more but smaller stick-slip events occurred for LH without PEG than MH with PEG. There existed a gradual increase in shear stress with loading time during LH without PEG, whereas this process was skipped and the stick-slip cycles were almost stable during MH with PEG. Inter-mainshock fault slips measured by a laser displacement transducer (LDT) at the fault edge also show a clear difference: insignificant fault slip was detected for LH without PEG (Supplementary Fig. 5a, c) whereas an accelerated fault slip was detected for MH with PEG (Supplementary Fig. 5b, d).

### Characteristics of foreshocks

We used the PZT array for analyzing seismic events that radiated waves with high enough signal-to-noise ratio (Supplementary Fig. 4c). Accordingly, all other processes not registered by the PZT array are termed aseismic in the current study. We located the hypocenters of the recorded seismic events and plotted only those considered as foreshocks (see “Methods” for the details of event detection and relocation). Their moment magnitude (*M*_w_) and relative time to the following mainshock are shown in Fig. 1e, f. Here the relative time means a normalized time-to-mainshock defined by t* = (*t*_m_−*t*)/(*t*_m_−*t*_pm_), where *t* is time, *t*_m_ is the time for the next mainshock and *t*_pm_ is the time for the previous mainshock. The number of identified foreshocks was 459 and 1120 for LH without PEG and MH with PEG, respectively, indicating a positive correlation between foreshock productivity and the amount of gouge. An even stronger correlation can be found between the distribution of foreshock hypocenters and that of gouge patches (Fig. 1a, e).

The statistical characteristics of the observed foreshocks differ between the two experiments: *b* value is 0.46 ± 0.03 and 0.33 ± 0.02 for LH without PEG and MH with PEG, respectively (Fig. 1g, Supplementary Table 2). Here we applied a tapered Gutenberg–Richter (TGR) model^29^ and estimated each *b* value by the maximum-likelihood method^29^. For the estimation of *M*_w_, we adopted the ball drop calibration technique proposed by McLaskey et al.^30^ (Supplementary Fig. 6). The smallest *M*_w_ estimated for the two experiments is −7.0. The observed foreshocks during the two experiments seem to follow a general scaling law for earthquakes (Supplementary Fig. 7). See “Methods” for the detailed methodology.

### Process leading up to the mainshock

**Fig. 2:**
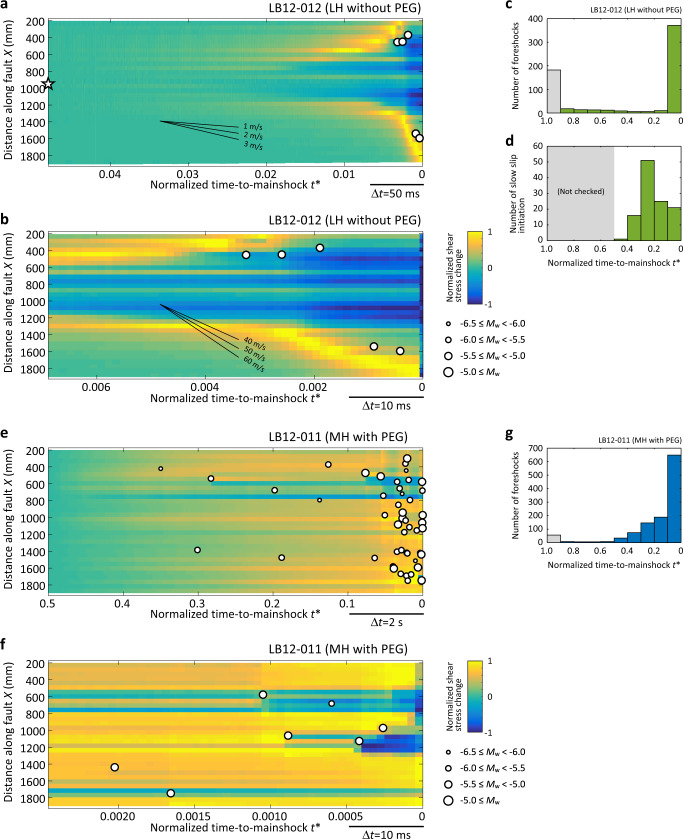
Spatiotemporal variations of shear stress and foreshock activity prior to the mainshock. **a** Typical evolutions of local shear stress and foreshocks just before a mainshock in LH without PEG. The color indicates the normalized shear stress change; the displayed shear stress in each location has been normalized by the maximum value after subtracting the initial value in the corresponding time period. See Fig. 1c, d for the absolute values. Open star indicates the time and location of the precursory slow slip initiation. **b** Zoom-in view of (**a**) for the last 50 ms. **c** Evolution of foreshock number in LH without PEG. **d** Histogram of the slow slip initiation times in LH without PEG. **e** Typical evolutions of local shear stress and foreshocks in MH with PEG. The displayed shear stress has been normalized in the same way as (**a**). **f** Zoom-in view of (**e**) for the last 50 ms. **g** Evolution of foreshock number in MH with PEG. Seismic events that occurred at t* larger than 0.9 (gray bars in (**c**) and (**g**)) were considered as aftershocks and were excluded from the analysis.

The dense strain and acoustic measurements reveal the detailed preparation process leading up to the mainshock. For LH without PEG, temporal increase and subsequent decrease in shear stress were observed (Fig. 2a, b; see also Supplementary Fig. 8 as a gray-scale version of Fig. 2). The boundary separating the increase and decrease of shear stress propagates outward with a slow but accelerated rate (from ~2 to ~50 m/s). These features indicate an underlying process known as precursory slow slip or preslip, common to some other laboratory experiments^7,8,10,17^. Further analysis by back-tracking the slip front^28^ shows that the slow slips initiated before the last quarter of the recurrence time (Fig. 2d) in many cases (32% of the total events). In contrast, most of the foreshocks occurred just before the mainshock, e.g., within 10% of the recurrence time (Fig. 2c).

**Fig. 3:**
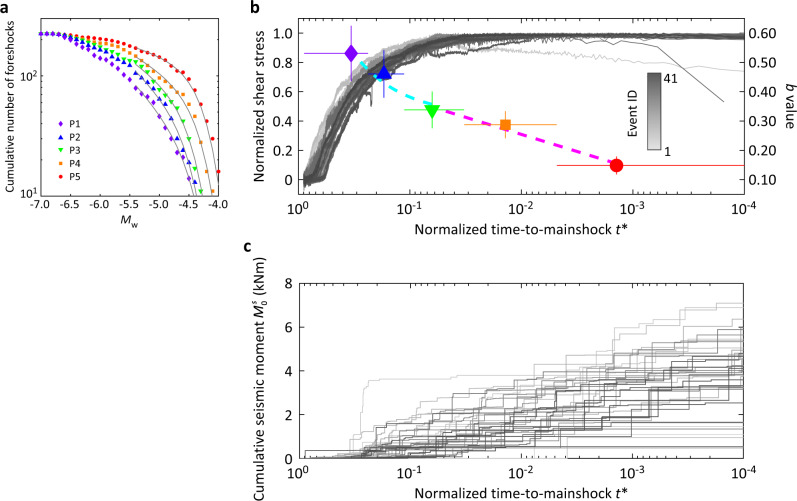
Evolutions of *b* value, macroscopic shear stress, and seismic moment in MH with PEG. **a** Frequency-size distribution of foreshocks for five windows of normalized time-to-mainshock t* (defined in Supplementary Table 3) in MH with PEG. Different colors and symbols represent data in different time windows. The width of each time window was determined to ensure an equal number of foreshocks therein (*N* = 224). The estimated *b* value and corner moment magnitude are listed in Supplementary Table 3. Gray lines show the best-fit curves to a TGR distribution. **b** Evolutions of macroscopic shear stress and *b* value in MH with PEG. The displayed shear stress has been normalized by each peak stress after subtracting each residual stress. Horizontal bar indicates the time window for calculating the related *b* value of foreshocks, while the horizontal location of colored symbol (following the same color code and symbol as in (**a**)) represents the corresponding median of t* of foreshock within each time window. Vertical bar indicates the standard error calculated with the maximum-likelihood method proposed by Kagan^29^ (see Supplementary Table 3). The dashed light blue and magenta curves represent the best fit between *b* value and t* during P1–P3 and P3–P5, respectively (see main text). **c** Evolution of cumulative seismic moment ${M}_{0}^{s}$ in MH with PEG. Gray scale of each curve follows that for Event ID in (**b**).

The corresponding preparation process for MH with PEG was quite different: a sudden decrease in local shear stress accompanied by foreshock occurrence was observed (Fig. 2e, f). Moreover, foreshocks occurred at an accelerated rate toward the mainshock, as can be observed from both the stacked results over all events (Fig. 2g) and the results for each individual event (Fig. 3c, Supplementary Figs. 9 and 10, note time is plotted on a logarithmic scale in Fig. 3c and Supplementary Fig. 9). Investigation of foreshock activity revealed a decreasing trend of *b* value toward the mainshock (Fig. 3a, b). In addition, the detailed decreasing behavior of *b* value evolved: following a linear trend with time *b* = 0.6806 t* + 0.3135 during P1–P3 whereas displaying a logarithmic trend *b* = 0.05069 log(t*) + 0.4877 during P3–P5. Time windows P1–P5 were defined in Supplementary Table 3.

## Discussion

Our study indicates that foreshock activity is closely connected to fault surface condition, because more foreshocks were generated under the condition with PEG and because the locations of foreshock hypocenters coincided well with the distribution of fault gouge (Fig. 1a vs.1e and1b vs.1f). Therefore, we suggest that gouge patches represent one type of asperities on the fault, which normally resist frictional slip but can radiate elastic waves when broken. As shown in Fig. 1g, *b* value is also affected by fault surface condition. Previous studies of seismicity reported a negative relation between *b* value and differential stress *τ*^21,23,24,31^. *b* value mapped on a laboratory fault also showed a low *b* value around asperities^18^. Our study shows that a heterogeneous distribution of PEG can cause a heterogeneous state of shear stress, which is featured by local stress concentrations around gouge patches (Fig. 1d). While fault gouge may also distinguish itself from host rocks in other aspects, such as elastic and frictional properties^32,33^, our analyses suggest that the gouge-induced stress heterogeneity should play the dominant role in determining the behaviors of foreshocks (see “Methods”). Specifically, the highly stressed gouge patches can shrink the size of rupture nucleation zone^2,34^ (keeping everything else the same), and hence can promote the generation of foreshocks there. Once nucleated, these events tend to grow bigger due to the availability of more energy (per unit area) and extra seismogenic area (Supplementary Fig. 3c, g), which could explain the relatively low *b* value for MH with PEG (Fig. 1g).

We note that some previous studies^35,36^ have reported a higher *b* value on a rough fault (presumably more heterogeneous) than on a smooth fault (presumably less heterogeneous), which is opposite to our finding (Fig. 1g). This discrepancy may be explained by the different ways of realizing fault heterogeneity. In those previous studies, heterogeneity most likely reflects the structural complexity of a fault network. The existence of fault stepover, bend, or discontinuity can easily stop the growth of a local rupture, and hence can lead to a high *b* value. In our MH case, heterogeneity is associated with a heterogeneous gouge distribution along a macroscopically continuous fault (Fig. 1b). Under this condition, stress amplitude can still fluctuate in space (Fig. 1d) but the failure planes of foreshocks will be restricted close to the pre-existing fault surface. Accordingly, one local event can expand its size or trigger another larger event by stress transfer projected along the same plane (Fig. 2e, f), which explains the relatively low *b* value as we observed. To clarify, the prepared fault in our study may be considered similar to a well-established plate interface at a subduction zone, where heterogeneity in the form of a patchy fault surface has been inferred from seismic and geodetic observations^37,38^.

**Fig. 4:**
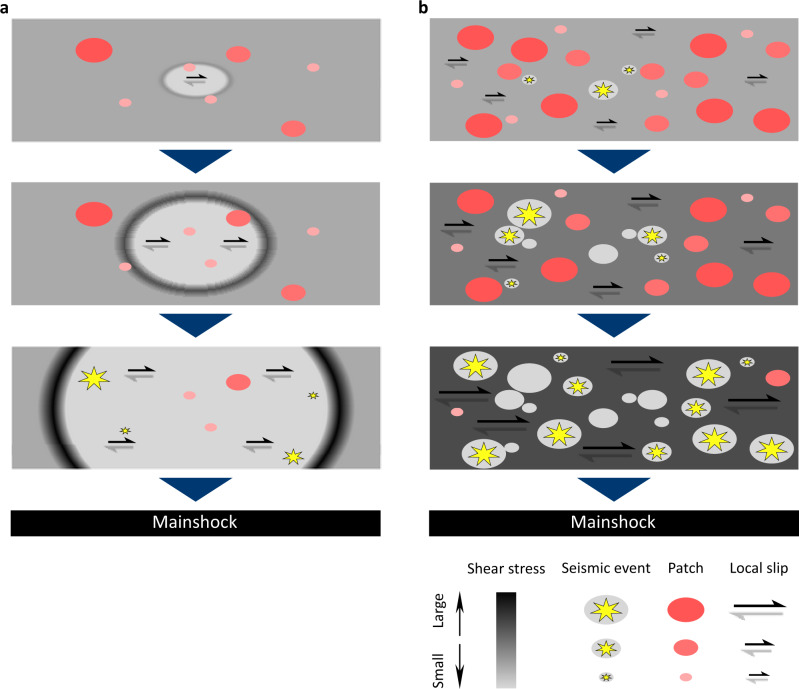
Schematic illustrations of two end-member earthquake preparation models. Earthquake preparation and related foreshock activity toward the mainshock driven by **a** a preslip process and **b** a cascade-up process. The fault surface contains fragile gouge patches (denoted by red ellipses) that can radiate seismic waves (denoted by yellow star) once they are broken. Magnitude of background shear stress is indicated by the intensity of the gray color.

In addition to shedding light on *b* value, our experimental results clearly demonstrate that different fault surface conditions lead to different types of preparation process toward the mainshock. Under the LH condition without PEG, typical precursory slow slip was observed (Fig. 2a, b). Slip measurement by the LDT at the fault edge indicates that the fault edge remained locked until slow slip reached there (Supplementary Fig. 5a, c). The spatiotemporal variations of local shear stress and foreshock activity further show that foreshocks were triggered during the later stage of slow slip, just after the passage of the slip front. We conclude that a gradually enhanced in situ stressing rate generated by an accelerated slip propagation, as confirmed by other experimental observations^8,39^, is required to trigger foreshocks under the LH condition without PEG (Fig. 4a). Therefore, foreshock activity, whose emergence depends on the availability and readiness of local asperities^40^, just represents a by-product of the ongoing slow slip under the LH condition without PEG. Meanwhile, the LH fault surface condition without PEG could facilitate the expansion and acceleration of the slow slip front. The concentration of foreshock occurrence just before the mainshock (Fig. 2c) should stem from such a situation. This implies a possibility for short-term prediction of the hypocentral location and in some cases the timing of the mainshock^40^. In nature, a similar scenario involving long-lasting aseismic slip, short-term slow slip transient, and immediate foreshocks has been observed before the 2011 Tohoku earthquake^25,41^.

The above scenario primarily driven by a single expanding slip patch was not observed under the MH condition with PEG. Although the LDT detected a seemingly smooth fault slip with an accelerated rate (Supplementary Fig. 5b, d), the near-fault strain gauge array showed that the actual slip was partitioned into numerous small patches and the local slip activity was essentially intermittent (Fig. 2e, f). We infer that the MH condition with PEG impedes the development of a well-connected large slip patch, and that the LDT-detected slip reflects a collective behavior (which tends to be smooth) of seismic/aseismic slips over many small fault patches^42^. The result shows a clear acceleration process of foreshock activity toward the mainshock, manifested by an increasing rate of seismic moment release (Fig. 3c and Supplementary Fig. 9a), an increasing foreshock magnitude (Fig. 2e, f), and an increasing foreshock number (Fig. 2g). Assuming that the LDT-detected slip is representative for the entire fault area, we further estimated the ratio of seismic moment ${M}_{0}^{s}$ to the total precursory moment release ${M}_{0}^{p}$, which showed a distribution around 4% for the stage right before the mainshock (Supplementary Fig. 9b). If correct, such low ratio would imply that most of the precursory moment release was actually occurring aseismically. The aseismic slip may occur spontaneously like the slow slip in the preslip model (but now distributed over numerous small fault patches). Alternatively, it may represent afterslip triggered by the intense foreshock activity. However, we must also bear in mind that ${M}_{0}^{p}$ could be overestimated due to the unjustified assumption of uniform slip over the entire fault area. Moreover, some very large foreshocks were not included in the PZT-based computation of ${M}_{0}^{s}$, because of the signal saturation or unclear wave arrivals. Taken together, it is likely that the above reported ratio of ${M}_{0}^{s}$ to ${M}_{0}^{p}$ could be underestimated. Future work by installing slip sensor arrays^43^ or by employing digital imaging correlation^44^ can better constrain the estimation of ${M}_{0}^{p}$.

The continuously decreasing *b* value prior to the mainshock (Fig. 3b) should be related to the acceleration process of foreshock activity. Decrease in *b* value prior to natural earthquakes has been reported^45,46^. Decreasing *b* value toward the mainshock was also reported in the laboratory and was explained by the increase in background shear stress^21,23^. The decreasing *b* value during P1–P3 should stem from the same mechanism, because both the decrease in *b* value and the increase in background shear stress show a linear trend with time during this time period (Fig. 3b and Supplementary Fig. 4b). Similarly, the relatively low *b* values (0.46 for LH condition and 0.33 for MH condition) with respect to a standard value around 1 can also be explained, because most of the foreshocks occurred just before the mainshock when the background shear stress had already become high. Alternatively, the relatively low *b* values could be derived from a smaller fractal dimension^47^, since in the current study foreshock locations were confined to the vicinity of a pre-existing fault, where two-dimensional effect could prevail (Fig. 1e, f). In nature, comparably low *b* value of 0.47 was reported around the upcoming mainshock hypocenter just before the 2011 Tohoku earthquake^45^.

Besides confirming an expected behavior of *b* value, Fig. 3b also shows an unexpected feature in that *b* value kept decreasing as log t* even after the background shear stress saturated at around P3. Moreover, the seismic moment released by foreshocks continued to increase during the same stage (Fig. 3c). We suggest that these evolutionary features during P3–P5 could result from the following cascade-up process via a positive-feedback mechanism and an increasing range of stress correlations (Figs. 2f and 4b): under a critical level of constant background loading, the local shear stress released by one foreshock is instantaneously redistributed at the residual fault area hosting other unbroken gouge patches; then the shear stress on the residual fault area is quickly increased every time a new and larger foreshock occurs, which can facilitate the triggering of more and even larger foreshocks in the future. We do not have enough resolution to reveal the exact triggering process, but we expect it could manifest as direct inter-event triggering^16,48^, correlate with rock damage^49^, or may be assisted by aseismic slip^17^. In any case, it appears that the evolutionary process during P3–P5 is mainly driven by the foreshock activity on gouge patches, which itself could interact with aseismic slip in the surrounding region. In a broad sense, the above interpretation is consistent with the concepts of critical point^50^, intermittent criticality^51^, and finite-time singularity^49^. In particular, the cumulative square root of foreshock-contributed seismic moment, called cumulative Benioff strain, can be well fitted to a power-law time-to-mainshock relation, with an exponent value similar to that observed for many natural earthquakes^52^ (Supplementary Fig. 10, Supplementary Table 4, see “Methods”). This suggests a possibility to predict the timing of the mainshock^49,50^. Although previous studies have further suggested a possibility to predict the magnitude or size of the mainshock^50^, testing the related hypothesis in the laboratory is not trivial, because properties of the simulated mainshocks (e.g., final rupture area, final slip, total source duration) are often influenced by artificial boundaries and the external apparatus^53,54^.

We have successfully reproduced both preslip and cascade-up processes toward the mainshock on a meter-scale laboratory fault. Especially, we have also demonstrated how the degree of fault heterogeneity can influence the selection between the two end-member preparation processes. It follows that the various debates or ambiguities on the earthquake preparation process^15,16,27^ may be resolved by incorporating in situ complementary observations (e.g., near-field acoustic, strain, and slip measurements), like what we have done in the laboratory. On the basis of in situ observations, we have further shown that several features of foreshock activity, such as timing, location, and statistical characteristics, can hint about the evolutionary process toward the upcoming mainshock. Therefore, monitoring the spatiotemporal pattern of seismicity and its evolution can help us to predict when and how the next major earthquake will occur.

Performing in situ observations for natural faults may seem difficult, but attempts and progress are being made. For the offshore region, seafloor observations implemented with multiple types of sensors are now available for the Nankai trough and the Japan trench^55^. For the inland region, the Southern California Earthquake Center has pushed for installing near-fault arrays of complementary sensors along major seismogenic faults, including the San Andreas fault^56^. Although the absolute scales differ between nature and laboratory, what really matter for earthquake prediction are the relative scales and the feasible instrumentations, thanks to the many power-law relations in seismology^42^ (e.g., the Gutenberg–Richter law, cumulative Benioff strain vs. time). In general, for studying the preparation process of a great earthquake (*M*_w_ $\ge$ 8), up to 100 Hz seismic recordings may be needed for analyzing foreshocks with a magnitude of completeness around 3 (ref. ^26^). Meanwhile, daily-to-monthly GPS recordings may be needed for capturing the acceleration onset of slow slip^41^, while hourly or high-rate (${\ge}$1 Hz) GPS recordings may be needed for analyzing the final evolution of slow slip right before the mainshock^57^. With these ongoing efforts, we believe our understanding of the preparation process of natural earthquakes will be improved as well.

## Methods

### Experimental setup

Supplementary Fig. 1 shows a schematic diagram of large-scale friction apparatus, which was installed on the large-scale shaking table at National Research Institute for Earth Science and Disaster Resilience (NIED), Tsukuba, Japan. The shaking table was used as a driving force to shear the simulated fault and it can generate fast (up to 1 m/s) and long (up to 0.4 m) fault slip. One rock specimen was vertically stacked on another rock specimen on the shaking table. The lower specimen was fixed by the frame and therefore moved with the shaking table. The upper specimen was supported by the reaction force bar, which is connected to the reaction force support on the outer floor isolated from the shaking table, so that the upper specimen stays against the frictional force on the simulated fault. The normal load was applied with three jacks above the upper specimen, and the amount of the load was measured with three load cells (TORD-S-400KN, Tomoe Research & Development, Ltd.) serially connected with each jack. The shear load was measured with a load cell (CLP-2MNS006, Tokyo Measuring Instruments Laboratory Co., Ltd.) installed between the eastern edge of the reaction force bar and the upper specimen. Relative distance between the upper and the lower specimens was measured with a laser displacement transducer, LDT (LK-G150, Keyence Corp.) installed at the western edge of the specimens. See Yamashita et al.^58^ for more detailed information about the apparatus.

### Specimen and fault surface

A pair of metagabbro blocks sampled from Tamil Nadu, south India was used as the rock specimens. The dimension of the upper specimen is 1.5-m long, 0.5-m wide, and 0.5-m high and that of the lower one is 2.0-m long, 0.1-m wide, and 0.5-m high. Therefore, the nominal contacting area (simulated fault area) is 1.5-m long and 0.1-m wide. Before the first experiment, every surface of the specimen was polished as flat as possible by a large-scale surface grinder in a rock company, so that the undulation over each surface is no more than 10 μm. Frictional slip during the experiment generated grooves and gouge on the simulated fault as shown in Supplementary Fig. 3. At the end of each experiment, the upper specimen was lifted up and then shifted back to its original position (without touching the lower specimen). Therefore, there was no reversed slip to disturb the generated gouge or grooves on the fault. Generated gouge was removed after each experiment except for LB12-010, whose loading rate and total amount of slip were 1 mm/s and 0.4 m, respectively. Therefore, the next experiment LB12-011 began with a lot of pre-existing gouge (PEG) on the fault as shown in Supplementary Fig. 3c. The conditions for other experiments can be found in Supplementary Table 1. Pictures of the fault surface were taken before and after each experiment (Supplementary Fig. 3). By differentiating the pictures before and after the gouge collection, the distribution of gouge can be estimated as shown in Fig. 1a, b. The procedure is the same as our previous study^58^. First, the differential picture was binarized with 0.1-mm resolution. Then, the fault surface area was divided into 5 mm × 5 mm elements and the number of counted data points was divided by all data points (50 × 50 = 2500) in each element. The obtained value should be proportional to the amount of gouge in the element and is defined as gouge density in this study. More details can be found in our previous study^58^. The *P* (*C*_*P*_) and *S* wave velocities (*C*_*S*_) of the specimen are 6.92 and 3.63 km/s, respectively. Detailed material parameters of the specimen can be found in Fukuyama et al.^59^.

### Local measurements

In order to monitor local phenomena during the experiments in detail, dense measurement arrays were installed along the fault (Supplementary Fig. 1b, c). Thirty-two three-component semiconductor strain gauges (SKS-30282, Kyowa Electronic Instruments Co., Ltd.) were glued on the northern side surface of the lower specimen. Signal from each component was individually processed by signal conditioner (CDA-700A/CDA-900A, Kyowa Electronic Instruments Co., Ltd.) and continuously sampled at 1 MHz with 16-bit resolution. Since each strain component of the three is individually recorded, normal and shear strain can be calculated at each location. For monitoring seismic activities, 64 shear mode piezoelectric transducers (PZTs) were glued on both side surfaces of the lower specimen. Resonance frequency of the used PZT (Fuji Ceramics Corp.) was 500 kHz. The signals were amplified 20 times by custom-made amplifiers (Turtle Industry Co., Ltd.) and continuously sampled at 10 MHz with 12-bit resolution.

### Determination of origin times and hypocenters of seismic events

Supplementary Fig. 4 shows seismic activities observed during the two experiments. In order to pick the time when a seismic event occurred, we first calculated the sum of squared amplitude over all PZT channels during a 0.1 ms time interval. Supplementary Fig. 4a, b shows the evolution of the calculated sum of squared amplitude as the selected time interval shifts. We next specified the event times by detecting local peaks of the calculated sum, and then pick up each time-window like Supplementary Fig. 4c. To locate hypocenters of seismic events, we picked arrival times of *P*/*S* waves by applying a STA/LTA (short-term average–long-term average ratio) signal detection technique. Distance between the hypocenter (*x*_0_, *y*_0_, *z*_0_) and the location of *i*-th PZT station (*x*_i_, *y*_i_, *z*_i_) can be written as follow:

1 $${D}_{i}=\sqrt{{({x}_{i}-{x}_{0})}^{2}+{({y}_{i}-{y}_{0})}^{2}+{({z}_{i}-{z}_{0})}^{2}}$$

We then searched the optimum parameters by a grid search technique so that the following *L*^2^ norm becomes minimum:

2 $${L}^{2}=\mathop{\sum }_{i=1}^{N}{\left({t}_{i}-{t}_{0}-\frac{{D}_{i}}{V}\right)}^{2}$$

where *N* is the number of PZT stations, *t*_*i*_ is the arrival time at *i*-th PZT station, *t*_0_ is the origin time, and *V* is the wave velocity. Here we treated the hypocenter, the origin time, and the wave velocity as unknown parameters and searched them under the assumption that the seismic events occurred on the fault surface (*z*_0_ = 0). To precisely locate the hypocenter, we picked the arrival times of *P* and/or *S* wave(s) again within a shorter time-window expected from the first located hypocenter and the origin time, and then we repeated the same procedure of grid search. Figure 1e, f shows the hypocenters of foreshocks finally located.

Now we can know when these seismic events occurred in each stick-slip cycle. From the time, we distinguished foreshocks from aftershocks: we investigated the normalized time-to-mainshock defined by t* = (*t*_m_−*t*)/(*t*_m_−*t*_pm_), where *t* is time, *t*_m_ is the time for the next mainshock, and *t*_pm_ is the time for the previous mainshock (Fig. 2c, g), and then we treated the seismic events whose normalized time-to-mainshock t* is larger than 0.9 as aftershocks, because they should occur just after the previous mainshock.

We next estimated a relative magnitude *M*_Lab_ of seismic event as an experiment-specific scale based on the amplitude of PZT outputs calibrated with distance^60^ as follows:

3 $$A=\sqrt{\frac{1}{K}\mathop{\sum }_{i=1}^{K}{\left(\frac{{r}_{i}}{10}{A}_{{i}_{\max }}\right)}^{2}}$$

4 $${M}_{{\mathrm{Lab}}}={\log }_{10}A$$

where *K* is the number of PZT stations, *r*_*i*_ is the distance between the hypocenter and *i*-th PZT station, and ${A}_{{i}_{\max }}$ is the maximum amplitude of seismic wave at *i*-th PZT station in volt. ${A}_{{i}_{\max }}$ was mainly derived from *S* wave.

### Estimation of moment magnitude

As a method for estimating the seismic moment *M*_0_ of a seismic event like acoustic emission observed in laboratory, a ball drop calibration technique has been proposed by McLaskey et al.^30^. In the current study, we followed their technique. Because this technique needs a record of ball drop impact as a reference source or empirical Green’s function, we carried out the ball drop tests and recorded the associated acoustic data using the same measurement system as the main experiments. A steel ball with a diameter of 3 mm was dropped from a height of 0.5 m onto the top surface of the lower specimen. The ball was dropped at 16 different locations (BD01-BD16) shown in Supplementary Fig. 1b and the drop procedure was repeated four times at each location. The four waveforms recorded at each PZT station were individually transformed to four Fourier spectra and were stacked together. Before the Fourier transform, the waveform record was tapered with a Blackman Harris window as done by McLaskey et al.^30^. We further averaged the Fourier spectra obtained at eight PZT stations adjacent to each ball drop location (the spectra at adjacent four stations were averaged in the case of ball drops at both edges BD01 and BD16). Supplementary Fig. 6a shows the averaged spectrum generated by the ball drop impact at BD01. In addition to these 16 reference spectra by the practical ball drop, we computed 15 extra reference spectra by virtual impact in the intermediate point between each ball drop location. For example, in the case of virtual ball drop between BD01 and BD02, we averaged the spectra by the ball drop impact at BD01 recorded by PZT02 and PZT34 as well as that at BD02 recorded by PZT03 and PZT35. As a result, we obtained 31 reference spectra by the ball drop impact in total. Since the location of the ball drop impact and the hypocenter of the seismic event to be calibrated should be close to each other, we used the reference spectra at the ball drop location closest to the hypocenter of the seismic event for each calibration. For this reason, we did not analyze those seismic events located outside of the PZT array. Typical uncalibrated spectra of three different foreshock events are shown in Supplementary Fig. 6a. The time window of waveform record was arranged so that both *P* and *S* waves are included, and the waveform record was tapered with a Blackman Harris window in the same manner as the ball drop record. These spectra were obtained by averaging the spectra recorded at four/eight PZT stations, which were the same stations used to calculate the relevant reference spectrum. In the case that no more than four individual records were available because of a trouble (e.g., saturation of signal), the moment magnitude of that event was not estimated. Supplementary Fig. 6b shows those source spectra calibrated by using the reference spectra of practical or virtual ball drop impact. As a scale factor to calibrate from the change in momentum to the moment, we used twice the average of the *P* and the *S* wave velocity by following McLaskey et al.^30^. It equals to 2(*C*_*P*_ + *C*_*S*_)/2 = 10.55 km/s for the metagabbro case. The flat level of spectrum at low frequency corresponds to the seismic moment. In order to robustly estimate the seismic moment *M*_0_ and the corner frequency *f*_c_ from the spectrum, we searched the optimum combination of *M*_0_ and *f*_c_ that minimizes the following residual *S*^2^ by a grid search technique:

5 $${S}^{2}=\mathop{\sum }_{i=1}^{J}{\{{\log }_{10}({S}_{i}(f))-{\log }_{10}({I}_{i}(f))\}}^{2}$$

where *J* is the number of the data points in the frequency domain, *S*_*i*_ is the calibrated source spectrum, and *I*_*i*_ is the omega-squared curve represented as *M*_0_/(1 + *f*^2^/*f*_c_^2^). Three best-fit curves are shown in Supplementary Fig. 6b. In this curve fitting, we ignored the data points whose signal to noise ratio at each frequency is <6 dB and those at troughs in the roll-off section (open square symbols shown in Supplementary Fig. 6b) for fitting precisely. We estimated the seismic moment of 274 seismic events (foreshocks and aftershocks) in LB12-011 and 226 ones in LB12-012 in total. Other seismic events could not be directly calibrated because their hypocenters were located outside of the PZT array or the quality of the associated source spectrum was not high enough. We then calculated the moment magnitude by using the following relation^61^:

6 $${M}_{w}=\frac{2}{3}{\log }_{10}({M}_{0})-6.067$$

Now we can compare the relative magnitude *M*_Lab_ obtained from the waveform amplitude with the calibrated moment magnitude *M*_w_. Supplementary Fig. 6c indicates that they are well correlated with each other. The best-fit relationship shown with a red line was obtained by the principal component analysis^62^ and is represented as

7 $${M}_{w}=1.0523{M}_{{{Lab}}}-5.8085$$

We used this empirical relationship to obtain *M*_w_ of the seismic event that could not be directly calibrated.

Supplementary Fig. 7 shows the relationship between the seismic moment and the corner frequency of the foreshocks in LB12-011 and LB12-012. The error bar represents uncertainty on the omega-squared curve fitting evaluated as follows: we searched the combinations of *M*_0_ and *f*_c_ whose residual *S*^2^ defined in Eq. (5) is <1.5 times of the minimum *S*^2^ for the best-fit curve, and defined the range from the minimum to the maximum of *M*_0_ or *f*_c_ that satisfies the above conditions as each uncertainty range. The dashed lines in Supplementary Fig. 7 show the constant stress drops 0.1, 1, and 10 MPa assuming the Brune’s model^63^. The amount of stress drop is estimated from the following relation:

8 $$\varDelta \sigma =\frac{7}{16}{M}_{0}{a}^{-3}$$

where *a* is the source radius and is represented as

9 $$a=2.34{C}_{S}/(2\pi {f}_{c})$$

As shown in Supplementary Fig. 7, most of the estimated stress drops of the foreshocks in both LB12-011 and LB12-012 fell between 0.1 and 10 MPa, which means that those foreshocks follow a general scaling law for seismic events in nature and laboratory^64^.

### Estimation of *b* value

It is well known that the frequency-size distribution of earthquakes obeys a power law called Gutenberg–Richter law given by log_10_*N*(*M*) = *A*−*bM*, where *N*(*M*) is the number of earthquakes greater than or equal to magnitude *M*, and *A* and *b* are constants^65^. The parameter *A* represents the number of earthquakes with magnitude larger than zero. The parameter *b* is usually referred to as the *b* value and it represents the relative size distribution. This law is also known to be applicable to seismic activity observed in laboratory^24^. The amount of *b* value corresponds to the slope of the frequency-size distribution plotted in the semi-log scale. It is also known that the frequency-size distribution has an exponential taper when there is an upper bound of seismic event^29^. Such distribution is called the tapered Gutenberg–Richter (TGR) distribution, and its cumulative complementary function Φ(*M*_0_) is given by

10 $$\Phi ({M}_{0})={({M}_{0}^{c}/{M}_{0})}^{\beta }\exp \left(\frac{{M}_{0}^{c}-{M}_{0}}{{M}_{0}^{co}}\right)\,{\mathrm{for}}\,{M}_{0}^{c}\le {M}_{0} < \infty ,$$

where ${M}_{0}^{c}$ and ${M}_{0}^{co}$ are the moment of completeness and the corner moment (in Nm), respectively. *β* is the index parameter of the distribution and $\beta =\frac{2}{3}b$. Since the fault area was finite, an upper bound of seismic event should exist in the current experiments. We applied the above distribution to the experimental data and then estimated *β* and ${M}_{0}^{co}$ with the maximum-likelihood method following the procedure proposed by Kagan^29^. We determined ${M}_{0}^{c}$ so that the misfit between the observed and modeled distributions becomes minimum^66^. The moment magnitude of completeness ${M}_{w}^{c}$ and the corner moment magnitude ${M}_{w}^{co}$, converted from the estimated ${M}_{0}^{c}$ and ${M}_{0}^{co}$, are listed in Supplementary Table 2. ${M}_{w}^{co}$ is −4.7 for LB12-012 and −4.3 for LB12-011. If we apply the Brune’s model with an assumed stress drop of 1 MPa, the corresponding source dimensions will be 73 and 116 mm in diameter, respectively, quite comparable to the width of the simulated fault (100 mm). Therefore, we conclude that it is appropriate to apply the TGR distribution to the current experimental data.

Generally, in order to examine temporal variation of *b* value toward the mainshock, time window including enough number of foreshocks is set and then *b* value in each time window is computed with shifting the window in a single seismic cycle^21,23^. However, the number of foreshocks observed during a single stick-slip cycle is not large enough even for the experiment MH with PEG. If one looks at the stick-slip cycle of the experiment MH with PEG (Supplementary Fig. 4b and Fig. 3b), one can find that the cycles are stationary almost over the whole experiment because the gouge generation process was skipped by the PEG. Therefore, we stacked the data with the normalized time-to-mainshock t* over different stick-slip cycles; we assumed that the fault state is the same with the normalized time-to-mainshock t* even among different stick-slip cycles. So, we made five data sets with five t*-time-windows, and then computed *b* value using each data set by fixing the moment magnitude of completeness at −5.5. The t*-time-window was determined so that 224 foreshocks are equally included in each window. Periods of each t*-time-window, *b* value, and the corner moment magnitude are given in Supplementary Table 3.

### Possible roles of fault gouge

To understand the differences between the LH fault without PEG and the MH fault with PEG, it is necessary to clarify the roles of fault gouge. It is known that fault gouge can exhibit reduced elastic moduli relative to host rocks^32^. One possible outcome is that fault gouge may reduce the critical nucleation zone size of foreshocks (often termed h*)^67^. However, one cannot just discuss reduced elastic moduli without mentioning the related length scale. In the current case, the naturally generated gouge layer is very thin: a few microns to tens of micron meters on average^32^. On the other hand, the source radius of the smallest foreshock in the well-calibrated range (*M*_w_ ~ −6.75, see Supplementary Figs. 6 and 7) is estimated to be 3.5 mm (Eqs. (6) and (8), assuming 1 MPa stress drop), about 100 times larger or more than the thickness of fault gouge. We acknowledge that there could be fluctuation in gouge layer thickness from one place to another, and hence we cannot rule out the possibility that in some places the gouge layer thickness can be comparable to the foreshock source dimension. But on average, we expect that the reduced elastic moduli of fault gouge did not help much in reducing h* of foreshocks, according to the non-local relation discussed in previous studies^32,67^.

Regarding the frictional properties of fault gouge, we did not perform any specific analysis for the current study. However, the observed coincidence between gouge patch location and foreshock location (Fig. 1) clearly suggests an overall velocity-weakening behavior for metagabbro fault gouge. This conclusion is also supported by our new experimental tests, in which metagabbro fault gouge of a uniform thickness was artificially distributed before the experiments. Specifically, Shimoda et al.^33^ estimated the macroscopic frictional properties of metagabbro fault gouge (3-mm thick before compaction), based on an isolated homogenized spring-slider model governed by a rate- and state-dependent friction law. They found that the rate- and state-dependent frictional parameter *b*–*a* (~0.001) was similar to that of metagabbro host rock^68^, whereas the other frictional parameter *L*_c_ (the critical slip distance) was about 2 orders of magnitude larger than that of metagabbro host rock. If we assume the above results are applicable to a gouge patch surrounded by other fault patches and can be extrapolated to an even thinner gouge layer (e.g., on the order of micron meter thick), then we would expect no significant difference in frictional properties between metagabbro gouge and metagabbro host rock, except for a somewhat larger *L*_c_ for the former because of the positive correlation between *L*_c_ and gouge layer thickness^69^. This suggests that, keeping everything else the same, fault condition with PEG could increase h* of dynamic ruptures^2^, and hence tends to impede the development of foreshocks.

Despite some uncertainties, our above analyses indicate that neither the elastic nor the frictional properties of fault gouge could satisfactorily explain the relative abundance of larger foreshocks under the condition with PEG (Fig. 1g). Then, we consider that the gouge-induced local stress concentration, as clearly revealed by our local strain measurements (Fig. 1d), must play a crucial role in causing those foreshocks as explained below. Earthquake nucleation model^2^ has predicted an inverse relation between h* and normal stress. It is also known that normal stress and shear stress are coupled along a frictional fault. Therefore, the local high normal and shear stresses around gouge patches could reduce h* of dynamic ruptures, and hence tend to promote the generation of foreshocks there. Theoretical expression of h* under a heterogeneous condition is still unknown. Nevertheless, a recent numerical study^70^ successfully simulated foreshock-like microseismicity on highly stressed patches embedded in an overall velocity-weakening fault section, which confirms the feasibility of local stress concentration for generating foreshocks.

In conclusion, although elastic and frictional heterogeneities also exist on the fault, the gouge-induced stress heterogeneity should play the dominant role in causing the foreshocks in MH with PEG. We note that the emergence of stress heterogeneity itself could stem from the property contrast between gouge patches and their surroundings, such that one should consider the interaction and feedback between different fault sections. For example, since the total amount of normal load was fixed during the current experiments, the local normal stress applied to the neighboring areas around the highly stressed gouge patches must be reduced. This, in turn, may explain the long-lasting aseismic slip (Supplementary Fig. 5b, d) observed in MH with PEG, because the corresponding h* could be quite large in those areas.

### Acceleration of seismic moment release

The seismic moment of each foreshock was estimated from the calibrated displacement spectra as explained in the section “Estimation of moment magnitude”. For the foreshock whose *M*_w_ was indirectly estimated from *M*_Lab_ and the relation (7), we used the relation (6) to calculate the seismic moment. Then we integrated the seismic moment for different foreshocks to obtain the cumulative seismic moment ${M}_{0}^{s}$, which monotonically increased as a function of time toward the mainshock (Fig. 3c and Supplementary Fig. 9a). To calculate the total precursory moment release ${M}_{0}^{p}$ over the entire fault area, we used the formula ${M}_{0}^{p}$ = *μD*_*p*_*S*, where *μ* is the shear modulus (39.3 GPa for metagabbro in the current study), *D*_*p*_ is the mean value of the precursory slip, and *S* is the fault area (1.5 × 0.1 m^2^). Here we assumed that the entire fault slipped uniformly by an amount equal to that measured by the LDT (Supplementary Fig. 5b). The total amount of *D*_*p*_ during the precursory stage was obtained from the average at the final stage just before the mainshock (10^−3^ < t* < 10^−4^).

After the above calculations, we could evaluate the ratio of ${M}_{0}^{s}$ to ${M}_{0}^{p}$ at the final stage just before the mainshock (Supplementary Fig. 9b). Care must be taken for interpreting the result shown in Supplementary Fig. 9, because the LDT measurement was most sensitive to the slip activity near the western edge of the fault (Supplementary Fig. 1a), and hence may not properly reflect the true state along other portions of the fault. In addition, the amount of ${M}_{0}^{s}$ could be underestimated due to the exclusion of some very large foreshocks. Those foreshocks often caused signal saturation of the nearby PZT sensors, or could not be located due to unclear wave arrivals. Taken together, we remark that the ratio of ${M}_{0}^{s}$ to ${M}_{0}^{p}$ shown in Supplementary Fig. 9 can be largely underestimated.

It is known that large/great earthquakes are sometimes preceded by an accelerated seismic activity as reviewed by Jaumé and Sykes^50^. It is also known that the cumulative square root of seismic moment, called cumulative Benioff strain, of those activities follows a power-law relation represented as

11 $$\sum {M}_{0}^{1/2}(t)={A}_{0}+B{({t}_{m}-t)}^{m}$$

where *t* is the time, *A*_0_, *B*, and *m* are the parameters describing the acceleration of phenomenon, and *t*_m_ is the time of mainshock. The exponent parameter *m* was reported to range from 0.1 to 0.55 for the case of natural earthquakes^52^. We also investigated the evolution of the cumulative Benioff strain toward the mainshock in the experiment MH with PEG. In order to obtain robust result, we focused on 14 accelerated sequences in which many (>20) foreshocks occurred during P3–P5. We applied the Nelder–Mead simplex algorithm^71^ to fit the relation (11) to the observed data and then estimated the parameters *A*_0_, *B*, and *m* following Bufe and Varnes^72^. Supplementary Fig. 10 and Supplementary Table 4 clearly demonstrate that the cumulative Benioff strains of foreshocks can be fitted to the relation (11) so well that the coefficient of determination *R*^2^ is 0.92 at least. Moreover, the estimated exponent parameters *m* are similar to those in natural earthquakes, which may suggest that the underlying mechanism of accelerated foreshock activity observed in the experiment MH with PEG is also similar to the one proposed for natural earthquakes.

## Supplementary information

Supplementary Information

## Data Availability

The source data (seismic event catalogs, preslip, and mainshocks) for reproducing the results of this study can be found in the Supplementary Information. The original laboratory data are available from the corresponding author upon request, because the file size is too large (4 TB in total). Source data are provided with this paper.

## Source data

Source Data

## References

[1] Ellsworth WL, Beroza GC (1995). Seismic evidence for an earthquake nucleation phase. Science.

[2] Dieterich JH (1992). Earthquake nucleation on faults with rate-and state-dependent strength. Tectonophysics.

[3] Lapusta, N. & Rice, J. R. Nucleation and early seismic propagation of small and large events in a crustal earthquake model. *J. Geophys. Res. Solid Earth* **108**, 10.1029/2001JB000793 (2003).

[4] Rubin AM, Ampuero J-P (2005). Earthquake nucleation on (aging) rate and state faults. J. Geophys. Res. Solid Earth.

[5] Noda H, Nakatani M, Hori T (2013). Large nucleation before large earthquakes is sometimes skipped due to cascade-up-Implications from a rate and state simulation of faults with hierarchical asperities. J. Geophys. Res. Solid Earth.

[6] Kaneko Y, Nielsen SB, Carpenter BM (2016). The onset of laboratory earthquakes explained by nucleating rupture on a rate-and-state fault. J. Geophys. Res. Solid Earth.

[7] Dieterich JH (1978). Preseismic fault slip and earthquake prediction. J. Geophys. Res..

[8] McLaskey GC, Kilgore BD (2013). Foreshocks during the nucleation of stick-slip instability. J. Geophys. Res. Solid Earth.

[9] McLaskey GC, Lockner DA (2014). Preslip and cascade processes initiating laboratory stick slip. J. Geophys. Res. Solid Earth.

[10] Ohnaka M, Kuwahara Y (1990). Characteristic features of local breakdown near a crack-tip in the transition zone from nucleation to unstable rupture during stick-slip shear failure. Tectonophysics.

[11] Okubo PG, Dieterich JH (1984). Effects of physical fault properties on frictional instabilities produced on simulated faults. J. Geophys. Res. Solid Earth.

[12] Latour S, Schubnel A, Nielsen S, Madariaga R, Vinciguerra S (2013). Characterization of nucleation during laboratory earthquakes. Geophys. Res. Lett..

[13] Ide, S. & Aochi, H. Earthquakes as multiscale dynamic ruptures with heterogeneous fracture surface energy. *J. Geophys. Res. Solid Earth* **110**, 10.1029/2004JB003591 (2005).

[14] Dodge DA, Beroza GC, Ellsworth WL (1996). Detailed observations of California foreshock sequences: implications for the earthquake initiation process. J. Geophys. Res. Solid Earth.

[15] Bouchon M (2011). Extended nucleation of the 1999 Mw 7.6 Izmit earthquake. Science.

[16] Ellsworth WL, Bulut F (2018). Nucleation of the 1999 Izmit earthquake by a triggered cascade of foreshocks. Nat. Geosci..

[17] McLaskey GC (2019). Earthquake Initiation From Laboratory Observations and Implications for Foreshocks. J. Geophys. Res. Solid Earth.

[18] Goebel, T. H. W. et al. Identifying fault heterogeneity through mapping spatial anomalies in acoustic emission statistics. *J. Geophys. Res. Solid Earth* **117**, 10.1029/2011JB008763 (2012).

[19] Lockner DA, Byerlee JD, Kuksenko V, Ponomarev A, Sidorin A (1991). Quasi-static fault growth and shear fracture energy in granite. Nature.

[20] Amitrano, D. Brittle-ductile transition and associated seismicity: experimental and numerical studies and relationship with the b value. *J. Geophys. Res. Solid Earth* **108**, 10.1029/2001JB000680 (2003).

[21] Goebel THW, Schorlemmer D, Becker TW, Dresen G, Sammis CG (2013). Acoustic emissions document stress changes over many seismic cycles in stick-slip experiments. Geophys. Res. Lett..

[22] Lei X, Kusunose K, Rao MVMS, Nishizawa O, Satoh T (2000). Quasi-static fault growth and cracking in homogeneous brittle rock under triaxial compression using acoustic emission monitoring. J. Geophys. Res. Solid Earth.

[23] Rivière J, Lv Z, Johnson PA, Marone C (2018). Evolution of b-value during the seismic cycle: Insights from laboratory experiments on simulated faults. Earth Planet. Sci. Lett..

[24] Scholz CH (1968). The frequency-magnitude relation of microfracturing in rock and its relation to earthquakes. Bull. Seismol. Soc. Am..

[25] Kato A (2012). Propagation of slow slip leading up to the 2011 Mw 9.0 Tohoku-Oki earthquake. Science.

[26] Kato A, Fukuda J, Kumazawa T, Nakagawa S (2016). Accelerated nucleation of the 2014 Iquique, Chile Mw 8.2 Earthquake. Sci. Rep..

[27] Tape, C. et al. Earthquake nucleation and fault slip complexity in the lower crust of central Alaska. *Nat. Geosci*. 10.1038/s41561-018-0144-2 (2018).

[28] Yamashita F (2018). Rupture preparation process controlled by surface roughness on meter-scale laboratory fault. Tectonophysics.

[29] Kagan YY (2002). Seismic moment distribution revisited: I. Statistical results. Geophys. J. Int..

[30] McLaskey GC, Lockner DA, Kilgore BD, Beeler NM (2015). A robust calibration technique for acoustic emission systems based on momentum transfer from a ball drop. Bull. Seismol. Soc. Am..

[31] Schorlemmer D, Wiemer S, Wyss M (2005). Variations in earthquake-size distribution across different stress regimes. Nature.

[32] Xu S, Fukuyama E, Yamashita F, Takizawa S (2019). Evolution of fault-interface Rayleigh Wave speed over simulated earthquake cycles in the lab: Observations, interpretations, and implications. Earth Planet. Sci. Lett..

[33] Shimoda, A., Yamashita, F., Fukuyama, E. & Watanabe, S. Friction parameter of metagabbro gauge obtained from a large-scale biaxial friction apparatus. in *SSJ Fall Meeting* Abstr. no S08-S20 (2020).

[34] Uenishi K, Rice JR (2003). Universal nucleation length for slip-weakening rupture instability under nonuniform fault loading. J. Geophys. Res. Solid Earth.

[35] Kwiatek G, Goebel THW, Dresen G (2014). Seismic moment tensor and b value variations over successive seismic cycles in laboratory stick-slip experiments. Geophys. Res. Lett..

[36] Goebel THW, Kwiatek G, Becker TW, Brodsky EE, Dresen G (2017). What allows seismic events to grow big?: Insights from b-value and fault roughness analysis in laboratory stick-slip experiments. Geology.

[37] Lay T (2012). Depth-varying rupture properties of subduction zone megathrust faults. J. Geophys. Res. Solid Earth.

[38] Yokota Y, Ishikawa T, Watanabe S, Tashiro T, Asada A (2016). Seafloor geodetic constraints on interplate coupling of the Nankai Trough megathrust zone. Nature.

[39] Xu S, Fukuyama E, Yamashita F (2019). Robust estimation of rupture properties at propagating front of laboratory earthquakes. J. Geophys. Res. Solid Earth.

[40] Ohnaka M (1992). Earthquake source nucleation: a physical model for short-term precursors. Tectonophysics.

[41] Mavrommatis AP, Segall P, Johnson KM (2014). A decadal-scale deformation transient prior to the 2011 Mw 9.0 Tohoku-oki earthquake. Geophys. Res. Lett..

[42] Ben-Zion Y (2008). Collective behavior of earthquakes and faults: continuum-discrete transitions, progressive evolutionary changes, and different dynamic regimes. Rev. Geophys..

[43] Yamashita, F., Fukuyama, E. & Xu, S. Foreshock activities controlled by slip rate on a 4-meter-long laboratory fault. *AGU Fall Meet*. Abstr. no MR11B-0034 (2019).

[44] Buijze L, Guo Y, Niemeijer AR, Ma S, Spiers CJ (2020). Nucleation of stick-slip instability within a large-scale experimental fault: effects of stress heterogeneities due to loading and gouge layer compaction. J. Geophys. Res. Solid Earth.

[45] Nanjo KZ, Hirata N, Obara K, Kasahara K (2012). Decade-scale decrease in b value prior to the M9-class 2011 Tohoku and 2004 Sumatra quakes. Geophys. Res. Lett..

[46] Schurr B (2014). Gradual unlocking of plate boundary controlled initiation of the 2014 Iquique earthquake. Nature.

[47] Aki, K. in *Earthquake Prediction: An International Review*, 4 (eds. Simpson, D. W. & Richards, P. G.) 566–574 (AGU, 1981).

[48] Helmstetter, A. & Sornette, D. Foreshocks explained by cascades of triggered seismicity. *J. Geophys. Res. Solid Earth* **108**, 10.1029/2003JB002409 (2003).

[49] Sammis CG, Sornette D (2002). Positive feedback, memory, and the predictability of earthquakes. Proc. Natl Acad. Sci. USA.

[50] Jaumé SC, Sykes LR (1999). Evolving towards a critical point: a review of accelerating seismic moment/energy release prior to large and great earthquakes. Pure Appl. Geophys..

[51] Ben-Zion, Y., Eneva, M. & Liu, Y. Large earthquake cycles and intermittent criticality on heterogeneous faults due to evolving stress and seismicity. *J. Geophys. Res. Solid Earth* **108**, 10.1029/2002JB002121 (2003).

[52] Ben-Zion, Y. & Lyakhovsky, V. Accelerated seismic release and related aspects of seismicity patterns on earthquake faults. *Pure Appl. Geophys.* **159**, 2385–2412 (2002).

[53] Kilgore, B. D., McGarr, A., Beeler, N. M. & Lockner, D. A. in *Fault Zone Dynamic Processes: Evolution of Fault Properties During Seismic Rupture* (eds. Thomas, M. Y., Mitchell, T. M. & Bhat, H. S.) 151–169 (AGU, 2017).

[54] Xu S (2018). Strain rate effect on fault slip and rupture evolution: Insight from meter-scale rock friction experiments. Tectonophysics.

[55] Aoi S (2020). MOWLAS: NIED observation network for earthquake, tsunami and volcano. Earth, Planets Sp..

[56] Ben‐Zion Y (2019). A critical data gap in earthquake physics. Seismol. Res. Lett..

[57] Ruiz S (2017). Nucleation phase and dynamic inversion of the Mw 6.9 Valparaíso 2017 earthquake in Central Chile. Geophys. Res. Lett..

[58] Yamashita F (2015). Scale dependence of rock friction at high work rate. Nature.

[59] Fukuyama E, Xu S, Yamashita F, Mizoguchi K (2016). Cohesive zone length of metagabbro at supershear rupture velocity. J. Seismol..

[60] Zang A (1998). Source analysis of acoustic emissions in Aue granite cores under symmetric and asymmetric compressive loads. Geophys. J. Int..

[61] Hanks TC, Kanamori H (1979). A moment magnitude scale. J. Geophys. Res..

[62] Jolliffe, I. T. *Principal Component Analysis* (Springer-Verlag, 2002).

[63] Brune JN (1970). Tectonic stress and the spectra of seismic shear waves from earthquakes. J. Geophys. Res..

[64] McLaskey GC, Kilgore BD, Lockner DA, Beeler NM (2014). Laboratory generated M-6 earthquakes. Pure Appl. Geophys..

[65] Gutenberg B, Richter CF (1944). Frequency of earthquakes in California. Bull. Seismol. Soc. Am..

[66] Clauset A, Shalizi CR, Newman MEJ (2009). Power-law distributions in empirical data. SIAM Rev..

[67] Kaneko, Y., Ampuero, J.-P. & Lapusta, N. Spectral-element simulations of long-term fault slip: effect of low-rigidity layers on earthquake-cycle dynamics. *J. Geophys. Res. Solid Earth* **116**, 10.1029/2011JB008395 (2011).

[68] Urata Y, Yamashita F, Fukuyama E, Noda H, Mizoguchi K (2017). Apparent dependence of rate- and state-dependent friction parameters on loading velocity and cumulative displacement inferred from large-scale biaxial friction experiments. Pure Appl. Geophys..

[69] Marone C, Kilgore B (1993). Scaling of the critical slip distance for seismic faulting with shear strain in fault zones. Nature.

[70] Schaal N, Lapusta N (2019). Microseismicity on patches of higher compression during larger-scale earthquake nucleation in a rate-and-state fault model. J. Geophys. Res. Solid Earth.

[71] Nelder JA, Mead R (1965). A simplex method for function minimization. Comput. J..

[72] Bufe CG, Varnes DJ (1993). Predictive modeling of the seismic cycle of the Greater San Francisco Bay Region. J. Geophys. Res. Solid Earth.

