# Supplementary material for: Two end-member earthquake preparations illuminated by foreshock activity on a meter-scale laboratory fault
Source: Nat Commun. 2021 Jul 14;12:4302. doi: 10.1038/s41467-021-24625-4 (PMC8280151; doi:10.1038/s41467-021-24625-4)
Supplement: Supplementary file 1 — Supplementary Information [file 41467_2021_24625_MOESM1_ESM.pdf]

Supplementary Information for

Two end-member earthquake preparations illuminated by foreshock activity on a meter-scale laboratory fault

Futoshi Yamashita[1]*, Eiichi Fukuyama[2,1], Shiqing Xu[3,1], Hironori Kawakata[4,1], Kazuo Mizoguchi[5,1], and Shigeru Takizawa[1]

[1]National Research Institute for Earth Science and Disaster Resilience, Tsukuba 305-0006, Japan
[2]Department of Civil and Earth Resources Engineering, Kyoto University, Kyoto 615-8530, Japan
[3]Department of Earth and Space Sciences, Southern University of Science and Technology, Shenzhen 518055, China
[4]College of Science and Engineering, Ritsumeikan University, Kusatsu 525-8577, Japan
[5]Central Research Institute of Electric Power Industry, Abiko 270-1194, Japan

*Corresponding author: Futoshi Yamashita
3-1 Tennodai, Tsukuba, Ibaraki, 305-0006, Japan
E-mail: yamafuto@bosai.go.jp

Supplementary Table 1. Experimental conditions.

Experiment ID	Loading rate, mm/s	Net amount of slip, mm	Gouge collection
LB12-001	0.01	7.0	Done
LB12-002	0.01	6.4	Done
LB12-003	0.01	1.8 (stopped by trouble)	Done
LB12-004	0.1	34.7	Done
LB12-005	1	386.2	Done
LB12-006	0.01	4.6	Done
LB12-007	0.01	6.7	Done
LB12-008	0.01	7.1	Done
LB12-009	0.1	34.9	Done
LB12-010	1	386.5	Not
LB12-011	0.01	7.2	Done
LB12-012	0.01	6.8	Done

Normal stress was fixed at 6.7 MPa in all the experiments.

Supplementary Table 2. *b* value and associated parameters.

Experiment ID	*b* value	M_w^c	M_w^{co}
LB12-012 (LH without PEG)	0.46±0.03	-6.4	-4.7
LB12-011 (MH with PEG)	0.33±0.02	-5.5	-4.3

M_w^c and M_w^{co} are the moment magnitude of completeness and the corner moment magnitude, respectively.

Supplementary Table 3. Five periods of normalized time-to-mainshock and the associated *b* value.

Period ID	From	To	*b* value	M_w^{co}
P1	0.87	0.24	0.53±0.09	-4.4
P2	0.24	0.11	0.46±0.08	-4.5
P3	0.11	0.033	0.34±0.06	-4.4
P4	0.033	0.0047	0.28±0.05	-4.3
P5	0.0047	0.000017	0.15±0.03	-4.2

Supplementary Table 4. Exponent value *m* and coefficient of determination R^2 for LB12-011 (MH with PEG).

Event ID	m	R^2
6	0.27	0.98
10	0.27	0.96
14	0.01	0.97
16	1.6×10^{-5}	0.97
18	0.11	0.97
19	0.13	0.94
20	0.10	0.96
23	0.38	0.95
24	0.50	0.96
28	0.14	0.97
29	0.28	0.98
35	0.17	0.92
37	1.1×10^{-6}	0.98
38	0.07	0.93

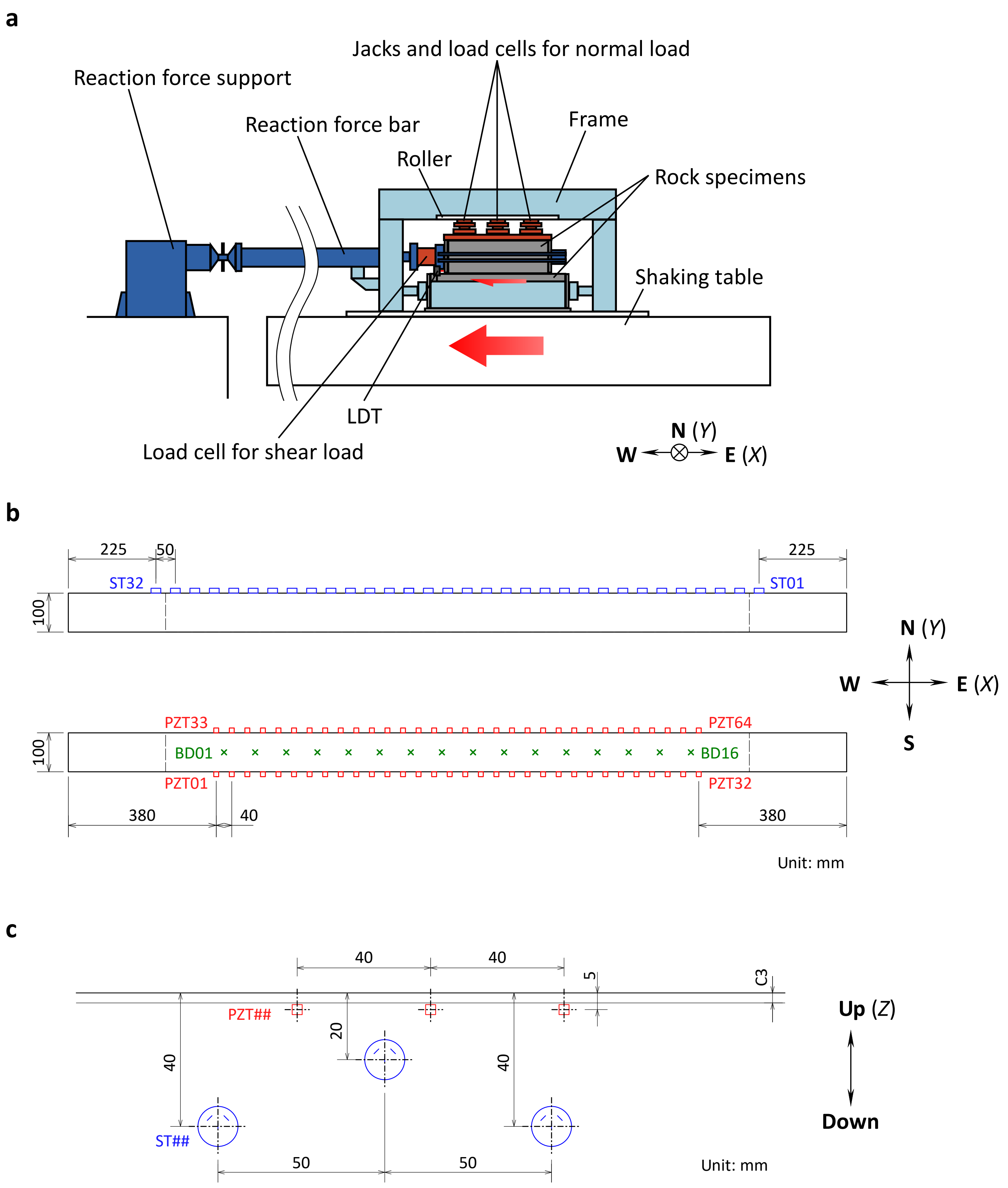

Supplementary Figure 1. Apparatus and observation. (a) Schematic diagram of large-scale friction apparatus at NIED. (b) Map view of the lower specimen indicating sensor and ball drop locations. Three-component strain gauges (ST) and piezoelectric acoustic sensors (PZT) were glued on the side surface of the lower specimen and are represented with blue rectangles and red squares, respectively. Green crosses (BD, which stands for ball drop) show the locations where ball for calibration was dropped. (c) Side view of sensor locations. Blue circle represents strain gauge and red square represents PZT. Even and odd IDs of strain gauge were installed at 20 and 40 mm away from the fault surface, respectively.

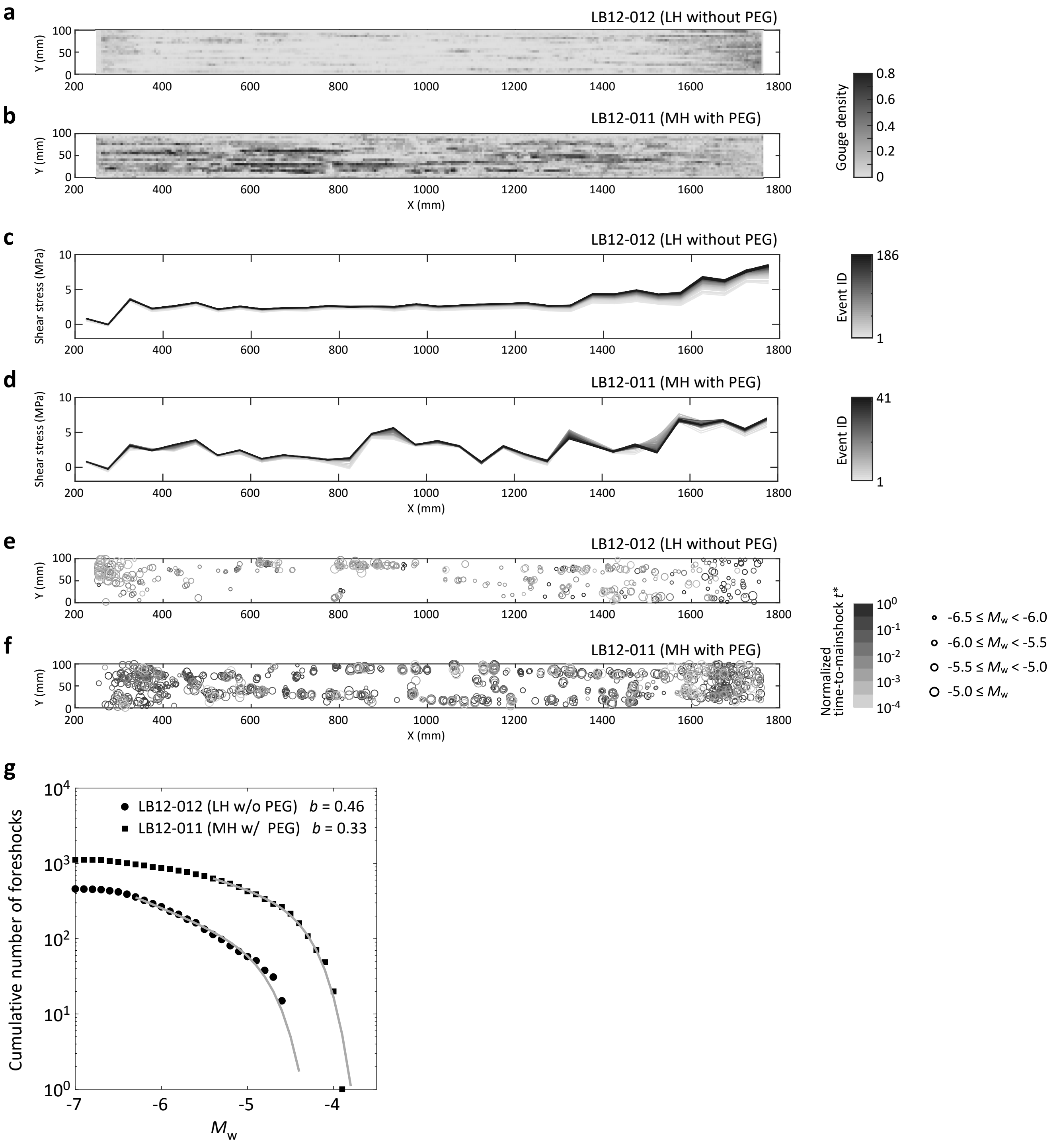

Supplementary Figure 2. Distributions of gouge, shear stress, and foreshocks. This is the gray-scale version of Fig. 1. Please refer to the caption of Fig. 1 for detailed explanation.

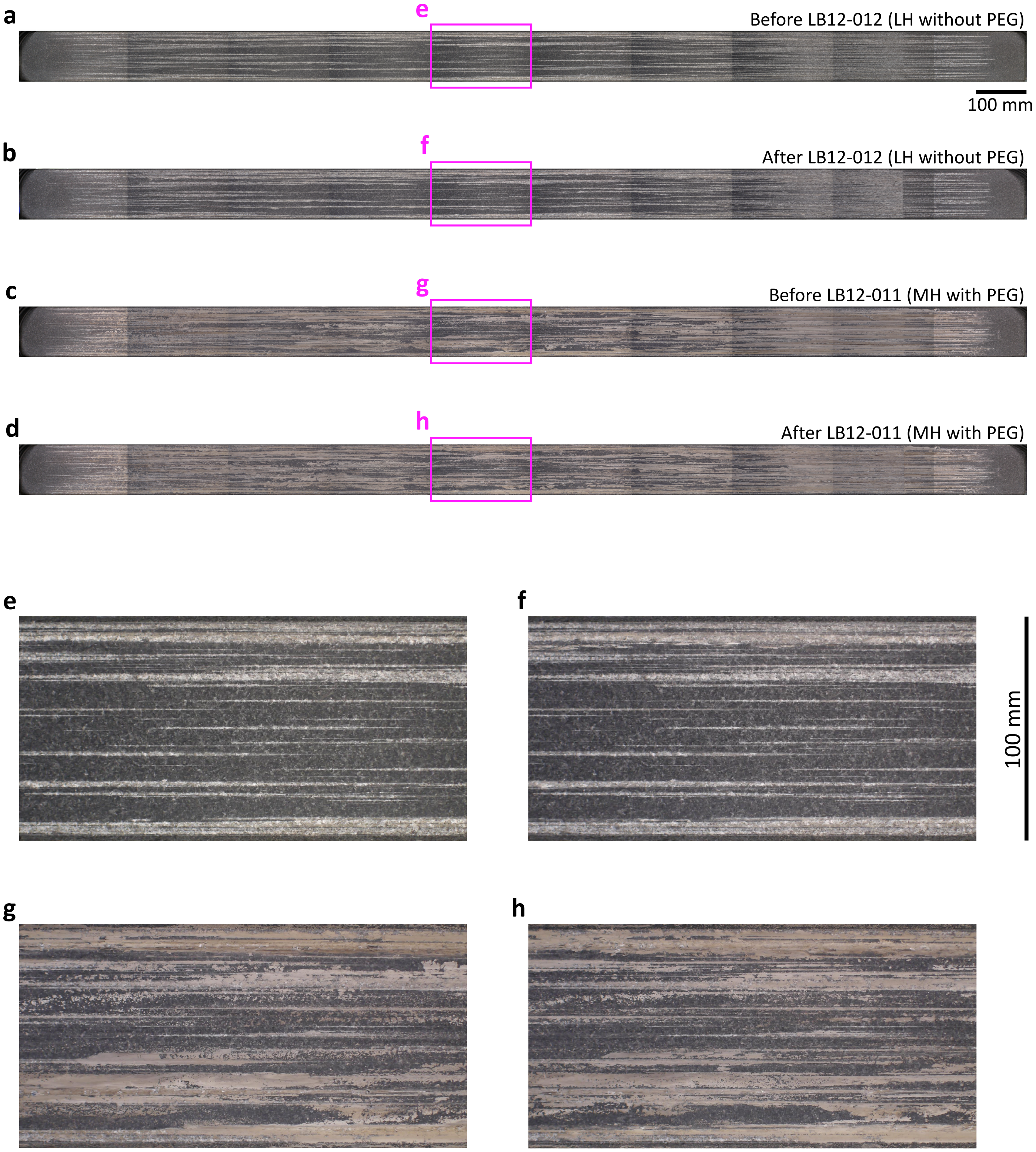

Supplementary Figure 3. Pictures of fault surface. (a) Picture taken just before the experiment LH without PEG, LB12-012. (b) Picture taken just after the experiment LH without PEG, LB12-012. (c) Picture taken just before the experiment MH with PEG, LB12-011. (d) Picture taken just after the experiment MH with PEG, LB12-011. (e) Zoom-in view of the area enclosed by the magenta rectangle in (a). (f) Zoom-in view of the area enclosed by the magenta rectangle in (b). (g) Zoom-in view of the area enclosed by the magenta rectangle in (c). (h) Zoom-in view of the area enclosed by the magenta rectangle in (d). Grooves and gouge generated by the frictional slip are recognized as white and gray-brown areas, respectively.

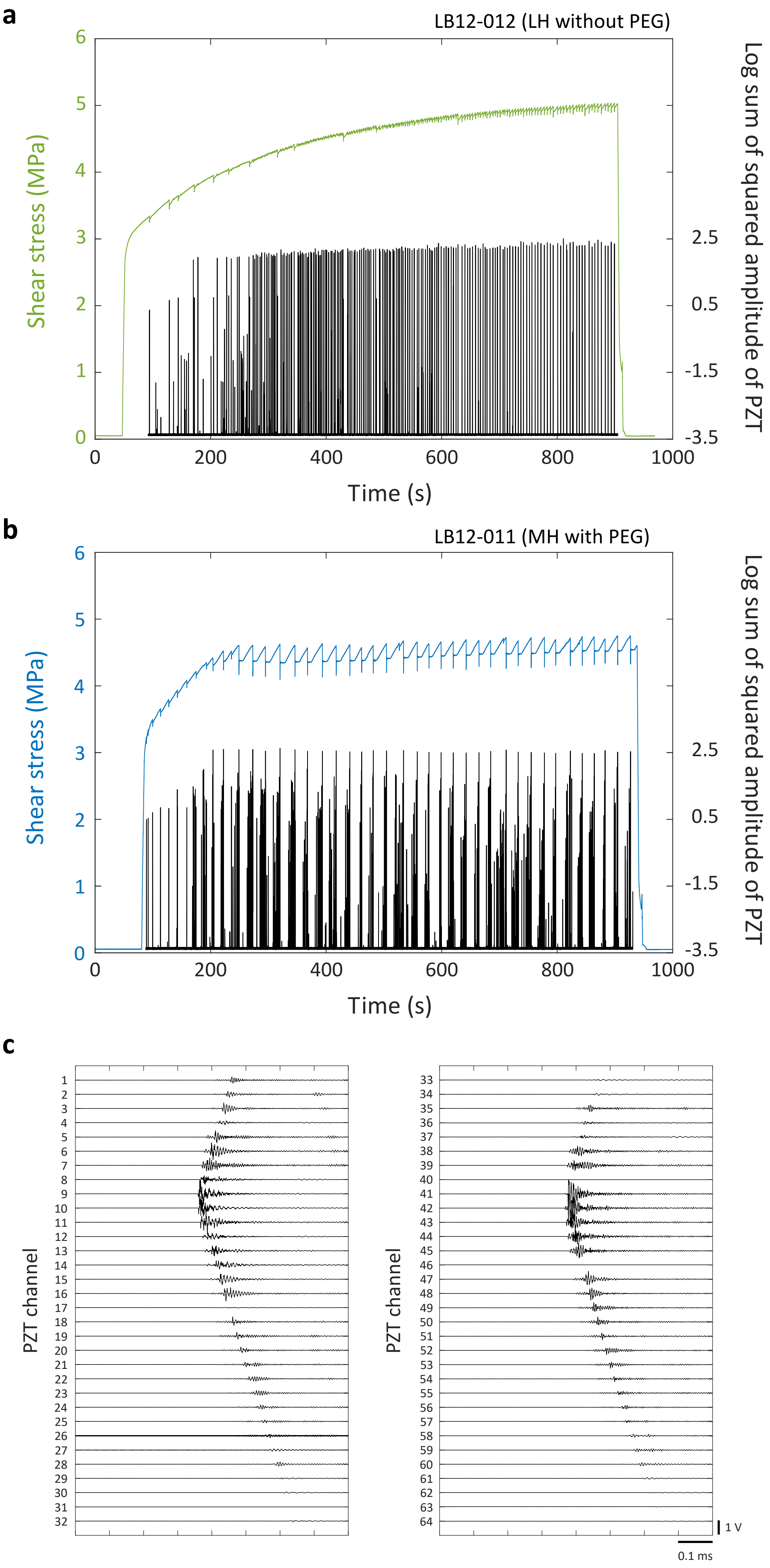

Supplementary Figure 4. Macroscopic shear stress and signals recorded by the PZT array. Shear stress and seismic activity as a function of time for the experiment (a) LB12-012 (LH without PEG) and (b) LB12-011 (MH with PEG). 186 and 41 stick slip events were observed and analyzed in LB12-012 and LB12-011, respectively. Squared amplitude of PZT output was accumulated over a duration of 0.1 ms. (c) Typical waveforms for a seismic event observed by the PZT array.

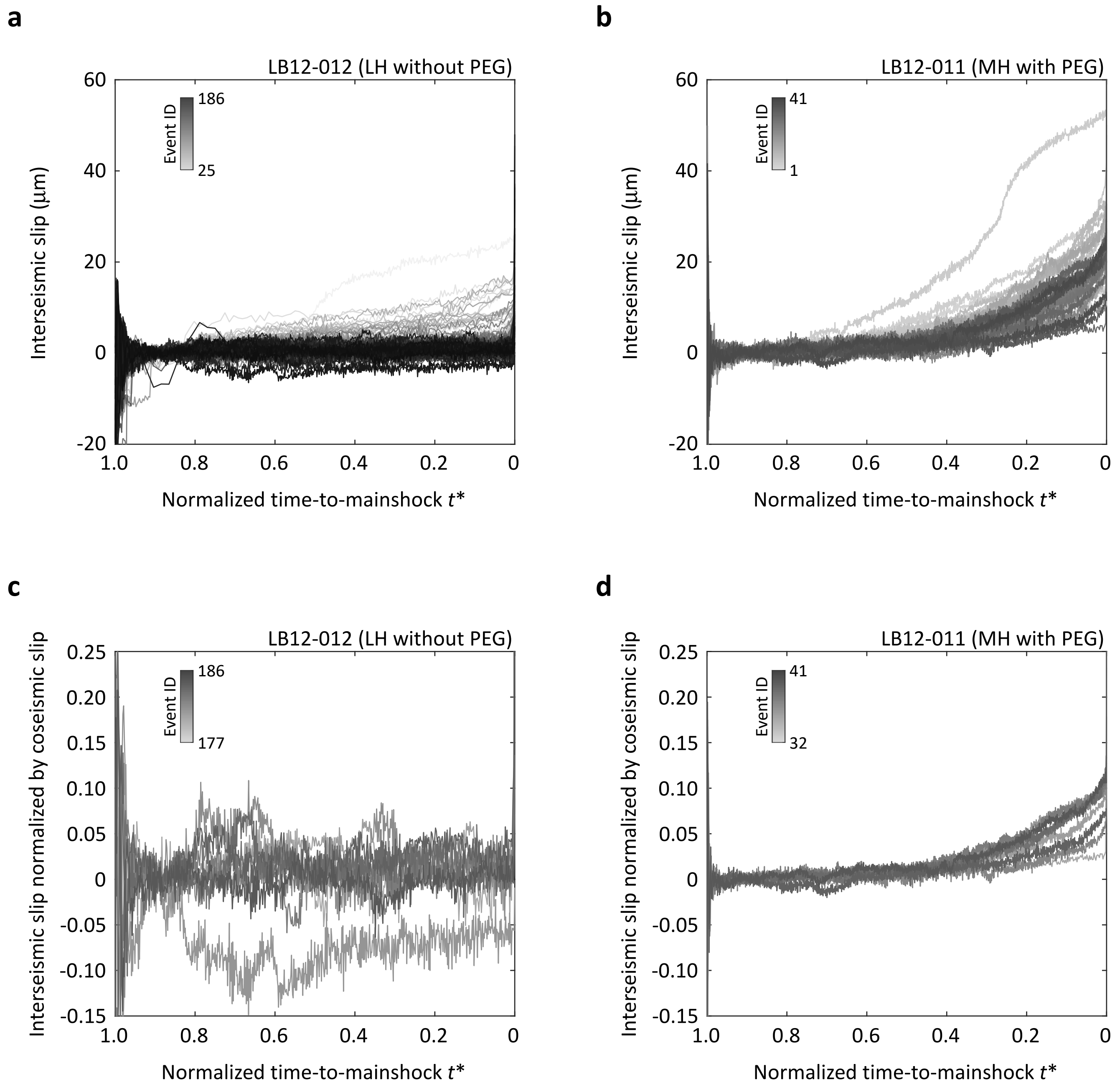

Supplementary Figure 5. Evolution of interseismic slip. (a) Evolution of interseismic slip (fault slip before the mainshock) in the experiment LH without PEG (LB12-012). The fault slip was measured with LDT shown in Supplementary Figure 1. The slip data for some earlier events (event ID less than 25) are not displayed because it seems that they did not reach a steady state. (b) Evolution of interseismic slip in the experiment MH with PEG (LB12-011). (c) Evolution of normalized interseismic slip (by the following coseismic slip) in the experiment LH without PEG. (d) Evolution of normalized interseismic slip in the experiment MH with PEG. To avoid complexity, only those slip data for the last ten events are displayed in (c) and (d).

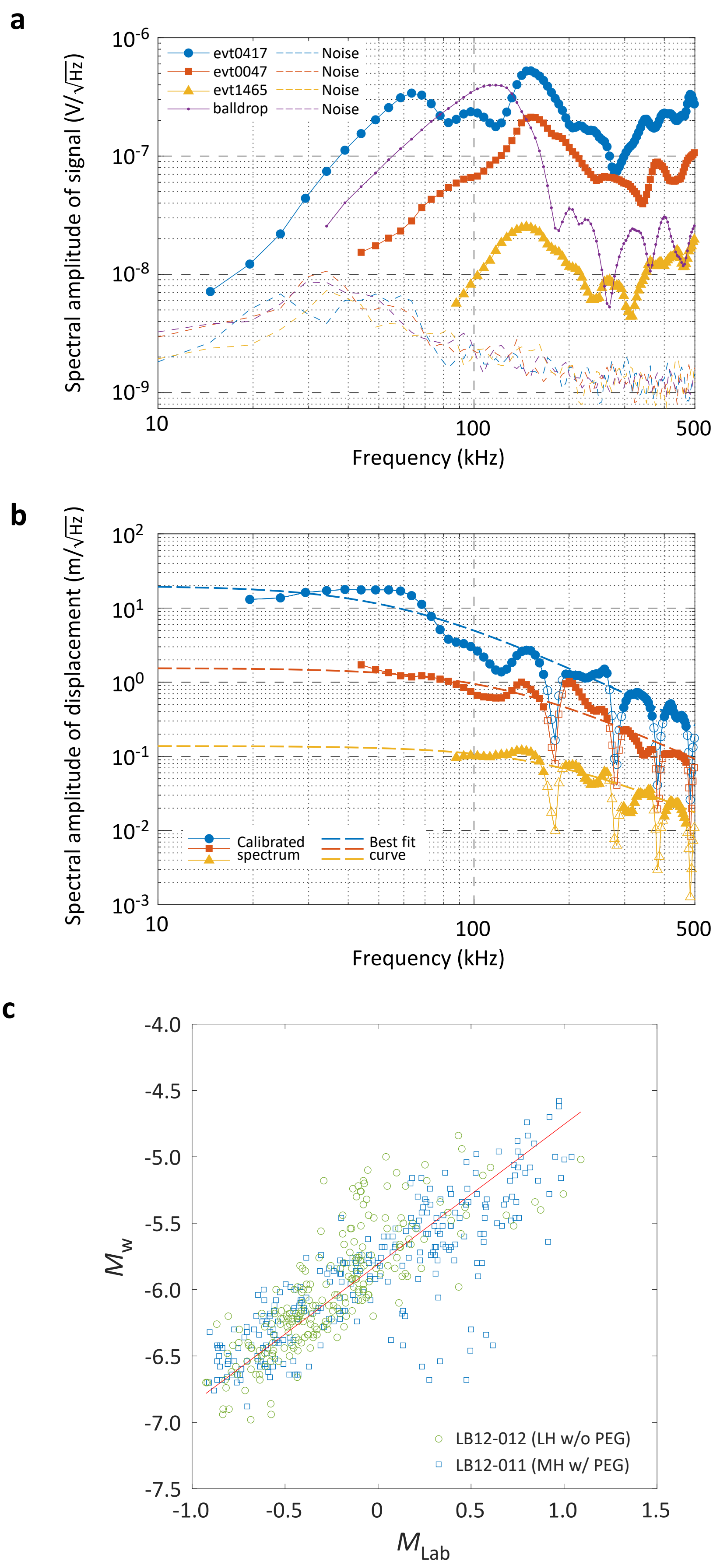

Supplementary Figure 6. Calculation of moment magnitude. (a) Typical uncalibrated amplitude spectra for three different foreshock events in LB12-011 and a spectrum for ball drop at BD01. Each noise spectrum is derived from the recordings before the *P* wave arrival. Only the data points whose S/N ratio is larger than 6 dB are plotted and used for further analysis. (b) Calibrated displacement spectra for the events shown in Supplementary Figure 6a. Dashed lines indicate the best-fit omega-squared curves. The data denoted by open square are ignored during curve fitting. (c) Comparison between lab-specific magnitude M_{Lab} and moment magnitude M_w of the seismic events (foreshocks and aftershocks) in both experiments. Red line shows the best-fit relationship calculated with the principal component analysis.

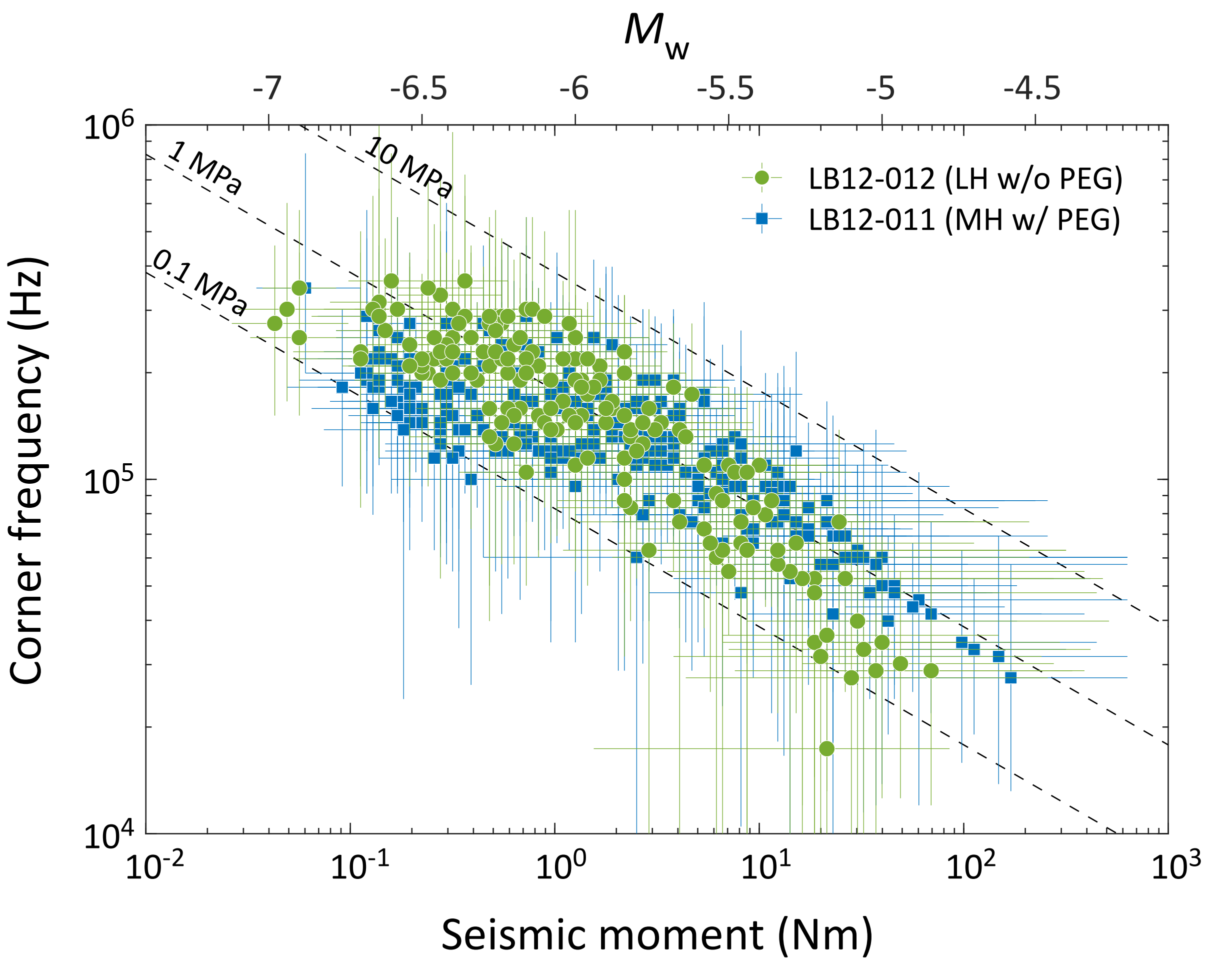

Supplementary Figure 7. Source properties of foreshocks. Corner frequency, seismic moment, and moment magnitude of the foreshocks in both experiments. The dashed lines indicate the predicted source properties by the Brune's model, with a constant stress drop of 0.1, 1, and 10 MPa. The error bars represent uncertainties during the omega-squared curve fitting (see Methods for the detail).

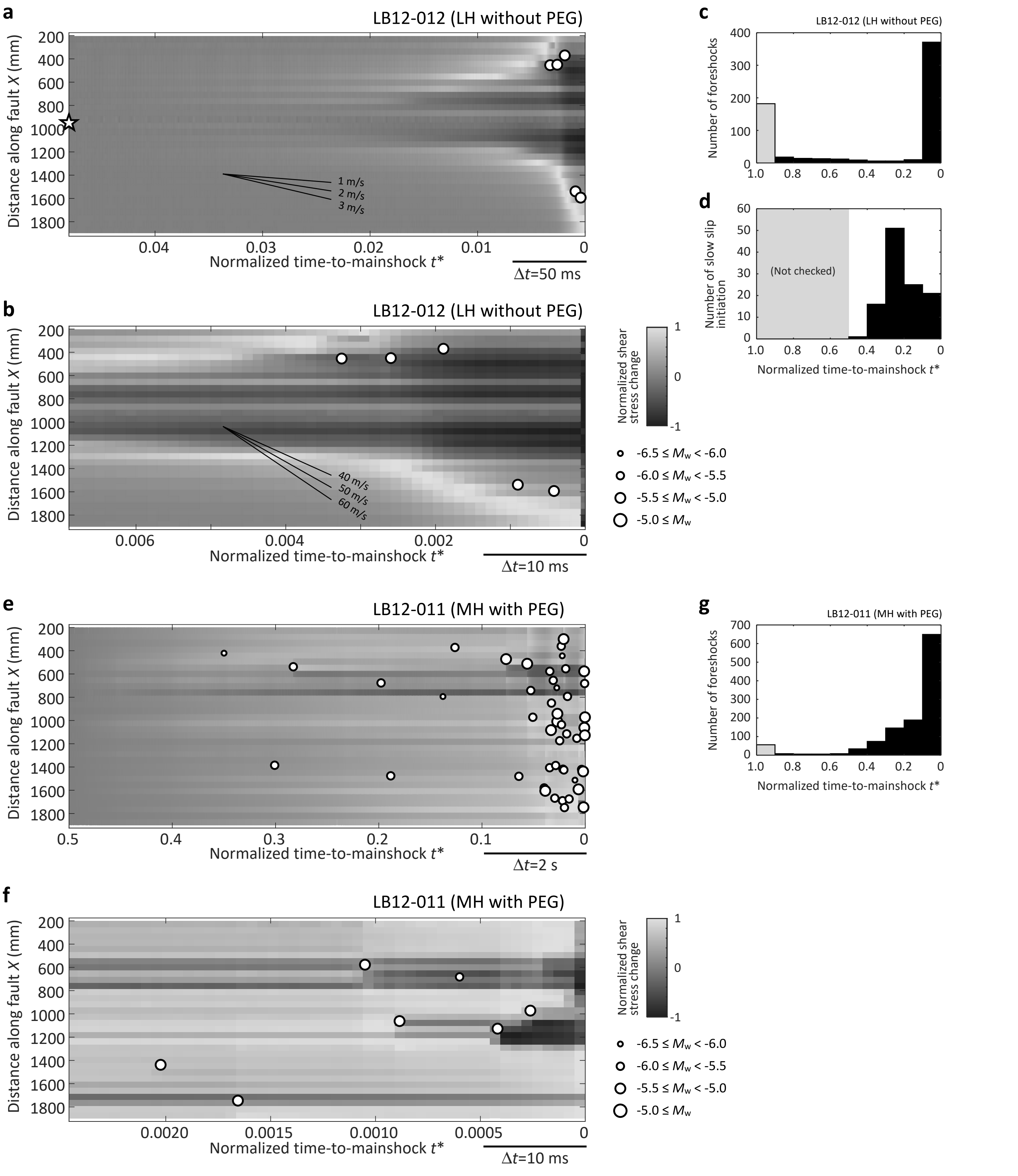

Supplementary Figure 8. Spatiotemporal variations of shear stress and foreshock activity prior to the mainshock. This is the gray-scale version of Fig. 2. Please refer to the caption of Fig. 2 for detailed explanation.

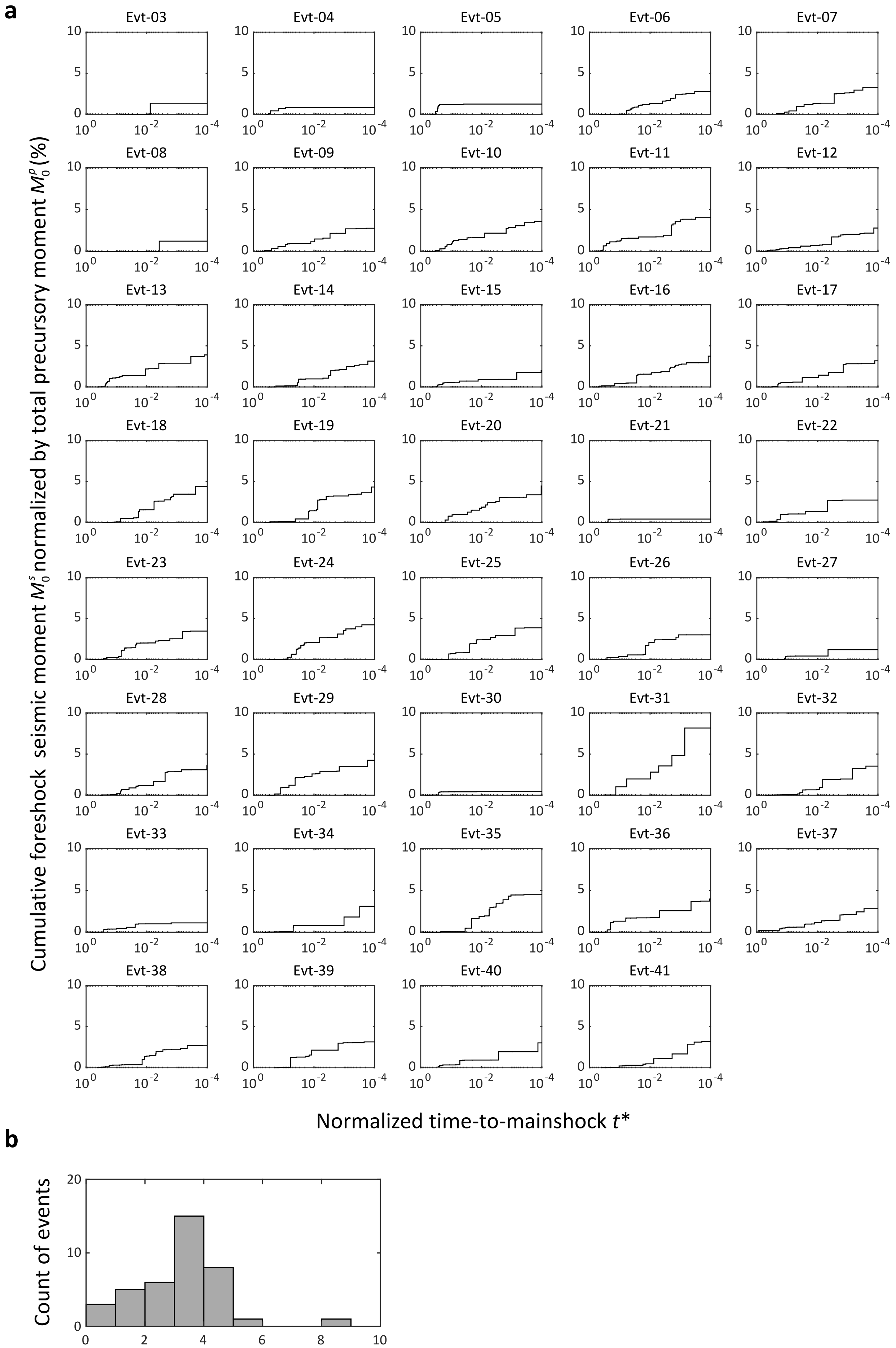

Supplementary Figure 9. Evolution of foreshock seismic moment. (a) Cumulative foreshock seismic moment M_0^s normalized by the total precursory moment release M_0^p as a function of normalized time-to-mainshock t^* in the experiment MH with PEG (LB12-011). (b) Histogram of the ratio of total M_0^s to M_0^p. See Methods for the detail.

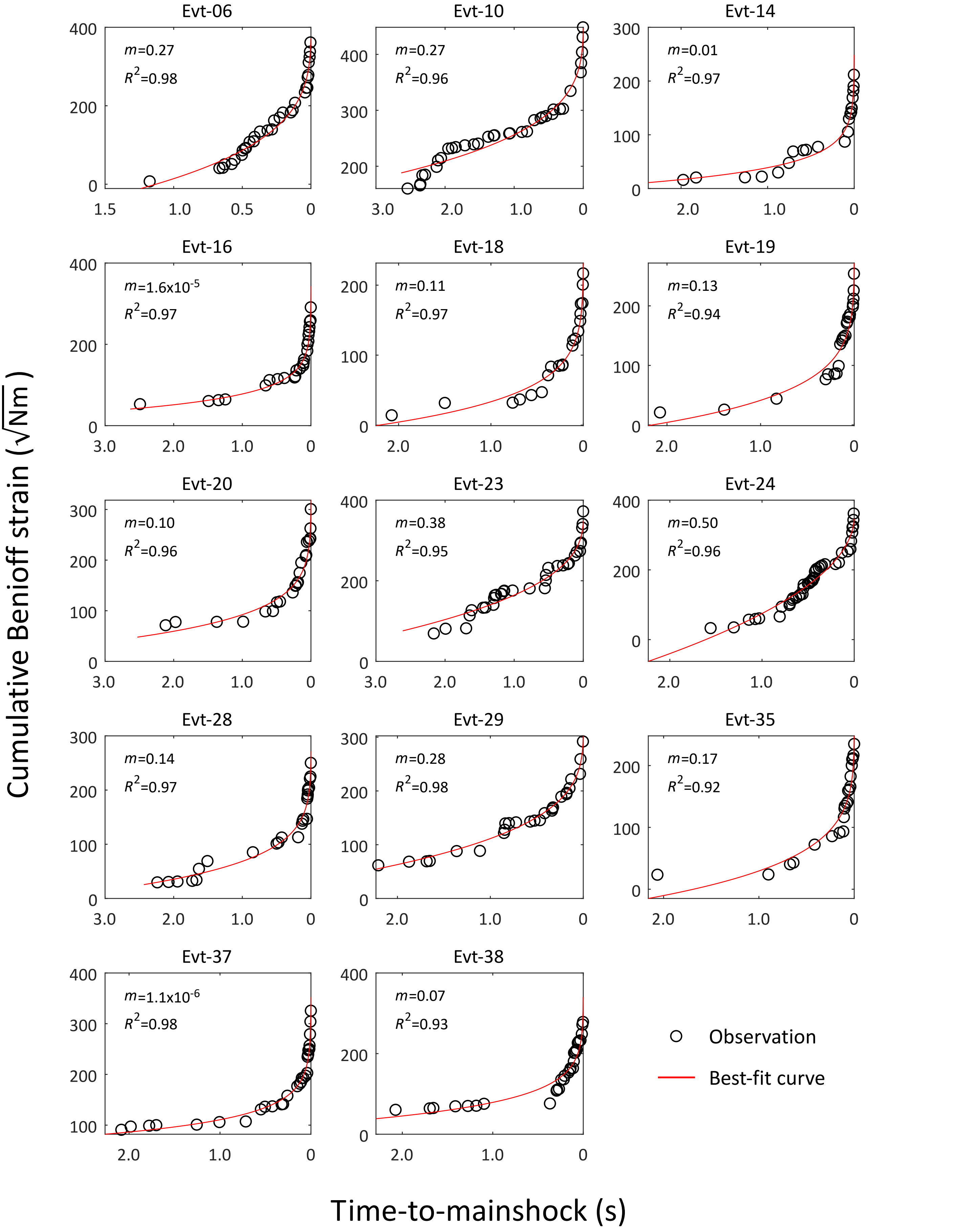

Supplementary Figure 10. Accelerated seismic moment release toward mainshock. Cumulative square root of seismic moment, called cumulative Benioff strain, as a function of time-to-mainshock at the final stage (P3-P5) in the experiment MH with PEG (LB12-011). Open circle represents cumulative Benioff strain calculated from the seismic moment of foreshocks. Red line represents the best-fit curve with a power law. The exponent value *m* and the coefficient of determination R^2 are displayed in each plotted area and Supplementary Table 4. Only the preparation process where more than 20 foreshocks occurred during P3-P5 is investigated. See Methods for the detail.
